# Predicting the combined effects of case isolation, safe funeral practices, and contact tracing during Ebola virus disease outbreaks

**DOI:** 10.1371/journal.pone.0276351

**Published:** 2023-01-17

**Authors:** Aliou Bouba, Kristina Barbara Helle, Kristan Alexander Schneider

**Affiliations:** 1 Hochschule Mittweida, University of Applied Sciences Mittweida, Mittweida, Germany; 2 Hochschule Merseburg, University of Applied Sciences Merseburg, Mittweida, Germany; 3 African Institute for Mathematical Sciences (AIMS), Limbe, Cameroon; Virginia Commonwealth University, UNITED STATES

## Abstract

**Background:**

The recent outbreaks of Ebola virus disease (EVD) in Uganda and the Marburg virus disease (MVD) in Ghana reflect a persisting threat of *Filoviridae* to the global health community. Characteristic of *Filoviridae* are not just their high case fatality rates, but also that corpses are highly contagious and prone to cause infections in the absence of appropriate precautions. Vaccines against the most virulent Ebolavirus species, the Zaire ebolavirus (ZEBOV) are approved. However, there exists no approved vaccine or treatment against the Sudan ebolavirus (SUDV) which causes the current outbreak of EVD. Hence, the control of the outbreak relies on case isolation, safe funeral practices, and contact tracing. So far, the effectiveness of these control measures was studied only separately by epidemiological models, while the impact of their interaction is unclear.

**Methods and findings:**

To sustain decision making in public health-emergency management, we introduce a predictive model to study the interaction of case isolation, safe funeral practices, and contact tracing. The model is a complex extension of an SEIR-type model, and serves as an epidemic preparedness tool. The model considers different phases of the EVD infections, the possibility of infections being treated in isolation (if appropriately diagnosed), in hospital (if not properly diagnosed), or at home (if the infected do not present to hospital for whatever reason). It is assumed that the corpses of those who died in isolation are buried with proper safety measures, while those who die outside isolation might be buried unsafely, such that transmission can occur during the funeral. Furthermore, the contacts of individuals in isolation will be traced. Based on parameter estimates from the scientific literature, the model suggests that proper diagnosis and hence isolation of cases has the highest impact in reducing the size of the outbreak. However, the combination of case isolation and safe funeral practices alone are insufficient to fully contain the epidemic under plausible parameters. This changes if these measures are combined with contact tracing. In addition, shortening the time to successfully trace back contacts contribute substantially to contain the outbreak.

**Conclusions:**

In the absence of an approved vaccine and treatment, EVD management by proper and fast diagnostics in combination with epidemic awareness are fundamental. Awareness will particularly facilitate contact tracing and safe funeral practices. Moreover, proper and fast diagnostics are a major determinant of case isolation. The model introduced here is not just applicable to EVD, but also to other viral hemorrhagic fevers such as the MVD or the Lassa fever.

## Introduction

After three cases of the Marburg virus disease (MVD) in Ghana [1, 2], the recent spread of the Ebola virus disease (EVD) in Uganda [3] marks the second outbreak of a filo virus in Africa in 2022. The Ebolavirus (EBOV) and the Marburgvirus (MARV) are the most prominent genera of the family of *Filoviridae*, which are non-segmented, negative-sense, single-strained RNA viruses [4, 5].

Both EBOV and MARV are highly contagious and lethal pathogens, classified as biosafety level 4 (BSL-4) agents and category A list pathogens [6], causing hemorrhagic fevers in humans and primates. Index cases emerge from zoonotic reservoirs [7]. Although the reservoirs have not been identified with certainty [8, 9], bats are suspected [10–12]. It is believed that EVD mostly spreads to humans by contact with primates, which have been infected through contact with infected bats [13]. Human-to-human transmission occurs by contact with blood and body liquids of symptomatic persons and infected corpses [14]. In particular, corpses of deceased persons are extremely contagious and both EVD and MVD have been reported to spread during unsafe funeral practices [15, 16]. Five EBOV species, four of which are known to cause EVD in humans [17], have been identified with substantially varying contagiousness and case fatality rates (25% to 90%) [18]. The current outbreak in Uganda is due to the *Sudan ebolavirus* (SUDV), which also caused a significant outbreak in Uganda in 2000/2001, and the first recorded EVD outbreak in 1976 in Sudan [17]. Because the international response was slow, the rather distinct and most prominent species, the *Zaire ebolavirus* (ZEBOV), was identified first. The ZEBOV is the most recurrent, contagious, and lethal species and was also the first one to be discovered in 1976 [18]. By far the majority of EVD outbreaks were caused by the ZEBOV followed by the SUDV with two larger and several minor outbreaks; all other EBOV species caused only minor outbreaks [19]. The current outbreak is particularly worrisome, because unlike previous EVD outbreaks an increasing number of cases occurs in the capital, i.e., in an urban rather than a rural area, where an outbreak is harder to control [20].

The Ebola epidemics from 2014–2016 in three West African countries (Guinea, Liberia and Sierra Leone) and from 2018–2020 in the Democratic Republic of the Congo (DRC), both caused by the ZEBOV, have been by far the largest recorded EVD outbreaks [4, 21–26], and substantially challenged global health-emergency management [27]. The outbreak from 2014–2016 amounted to 28,600 cases and 11,325 deaths, yielding a case fatality rate of 39.59% [12, 28–30]. During the outbreak from 2018–2020 in two Eastern provinces of DRC 3,453 cases and 2,273 deaths were recorded [25], resulting in a case fatality rate of 67% [23, 28]. The severity of the outbreak in DRC was fuelled by armed conflicts in the affected areas [25, 31]. The government and international community had only limited access to the affected areas, which had only poor-quality health centers, thereby increasing mortality [26, 32].

Regarding the pathogenesis of EVD, the incubation period ranges from 2 to 21 days [33, 34]. (Notably, the same range is commonly reported for the MVD, but was recently found to be an underestimate [2]). EBOV infects many types of body cells, and thereby produces EBOV glycoproteins that attach to the inside of blood vessels, rendering them to be more permeable [5, 35, 36]. The increased permeability causes the blood vessels to leak blood [37]. EBOV also invades other body parts and organs (liver, spleen, kidney, and brain), which can lead to organ failure and death [38]. The virus also counteracts the host’s natural defense system, by infecting immune cells [4]. Although it is unclear whether survival of EVD confers permanent immunity (because this can only be ascertained during large epidemic outbreaks), evidence suggests long-lasting immunity after recovery [39, 40].

The symptomatic phase of EVD is characterized by a sudden rise in temperature, weakness, muscular pain, headache, and pain in the throat during days 1–3 [18]. During days 4–7 cutaneous eruptions, renal and hepatic insufficiency, internal and external bleeding can occur after the appearance of vomiting and diarrhea [41]. Finally, infected individuals may present with confusion and may exhibit signs of internal and/or visible bleeding, potentially progressing towards coma, shock, and death during days 7–10 [42].

So far, no approved drug treatment exists for the EVD. However, some treatments are associated with improved clinical outcomes [43–46]. Re-hydration therapy and infusions are known to reduce the severity of symptoms [47–50]. Since 2017 three vaccines against the ZEBOV species have been approved [26, 51–55]. Pre- and post-exposure vaccination was a cornerstone of disease management during the 2018–2020 EVD outbreak in DRC [56]. For the recent outbreak of the SUDV in Uganda, the efficiency of current vaccines is unclear [57, 58], but it is assumed that the current vaccines are ineffective [59]. Notably, there exist three vaccine candidates in phases I studies and several more in pre-clinical trials [60]

Contact tracing and quarantine strategies are the most important pillars of managing EVD outbreaks [61, 62]. Moreover, safe funeral practices are fundamental [27, 63–65]. These are challenges in the beginning of an outbreak, because of a lack of on-site infrastructure to diagnose *Filoviridae* by PCR [66]. In fact, the index case in the recent MVD outbreak was unsafely buried because the virus was diagnosed *postmortem* [2]. Another problem arose from the underestimated incubation period [67, 68]. Namely, the secondary cases developed symptoms after they completed a 21-days quarantine. Due to the similarities of MVD and EVD, the same challenges also apply to the latter. Furthermore, contact tracing and isolation as primary control strategy present significant logistic and economic strains on the public health systems in low income countries [69].

**Fig 1:**
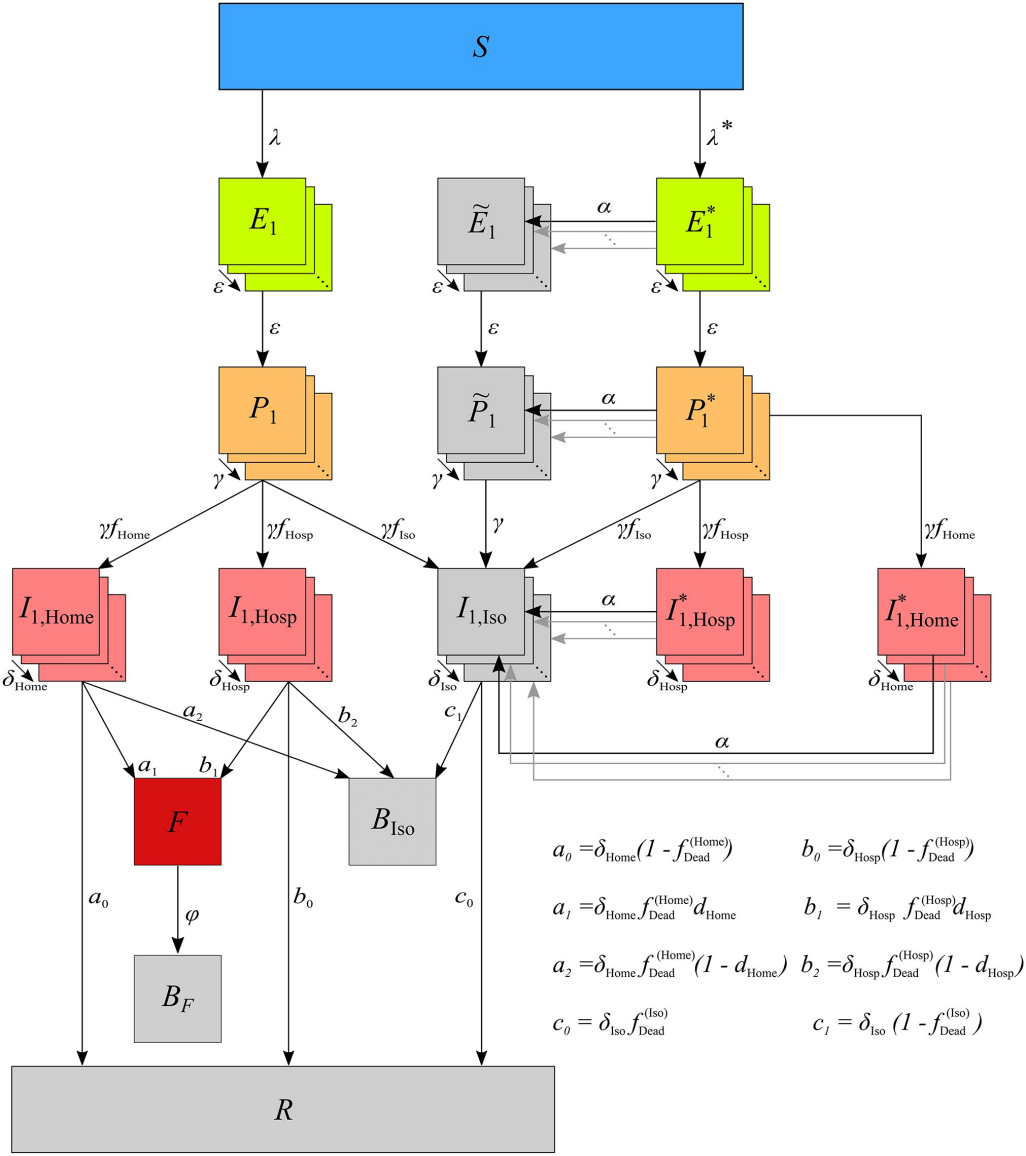
Model. Stages are depicted as boxes, arrows show transition rates. The entire population is grouped into the susceptibles (*S*), the infected which are further classified into the latent (*E*), the prodromal, (*P*), the fully infectious at home, (*I*_Home_), in hospital, (*I*_Hosp_), and in isolation, (*I*_Iso_), and the recovered (*R*). The dead are classified into those awaiting an unsafe Funeral (*F*)—still infectious, after funeral (*B*_*F*_), or buried safely (*B*_Iso_). Trace back is modeled through the force of infection for infections subject to trace back λ*, resulting in individuals in transient stage (E*, P*, *I*^(*, Home)^, *I*^(^*^, Hosp)^) who will get traced back later, and those ($\tilde{E},\tilde{P},I_{\text{Iso}}$) who have already been traced back or diagnosed—these are isolated; λ and *E*, *P*, *I*_Home_, *I*_Hosp_ describe infections not subject to trace back. The rates *ε*, *γ*, *δ*_Home_, *δ*_Hosp_, *δ*_Iso_ describe the progression of the infections. *α* is the rate of successful trace back, *φ* the one of funerals, *d*_Home_, *d*_Hosp_ indicate the fractions of safe funerals of non-diagnosed individuals. *f*_Home_, *f*_Hosp_, *f*_Iso_ are the fractions of fully infectious in different treatment, where $f_{\text{Dead}}^{\left(\text{Home}\right)},f_{\text{Dead}}^{\left(\text{Hosp}\right)},f_{\text{Dead}}^{\left(\text{Iso}\right)}$ are the related death rates.

Here, we introduce a predictive model to study the effect of case isolation, safe funeral practices, and contact tracing during EVD epidemics on disease mortality. To the best of our knowledge, this is the first model of EVD which studies the combined effect of safe funeral practices and contact tracing together. The model is a complex extension of an SEIR-type model (see Fig 1 for an illustration). Model parameters, which have mainly been estimated for the ZEBOV (cf. [70]), are chosen from the literature adjusted such that the dynamics reflect the situation in rural areas in Africa. The model is *per se* also applicable to the MVD, however, past outbreaks were relatively small compared to EVD outbreaks, so a deterministic approach is questionable for the MVD. In the main text, the model is first introduced verbally. A concise mathematical description for readers interested in the technical details is available as Supporting Material. The model is implemented in Python and available at https://github.com/Maths-against-Malaria/Ebola. Outcomes of numerical investigations are presented in the result section.

## Methods

The predictive model introduced here is a complex extension of an SEIR-type model. Because of its complexity, the model and its basic notation are described here only verbally, guided by the flow chart presented in Fig 1. Readers with a strong mathematical background, who are interested in the model equations shall feel free to skip this section and directly move to the formal description in S1 Appendix, where the model compartments and parameters are first introduced in a systematic manner, followed by the resulting set of differential equations.

### Basic model compartments

We assume a population of size *N*. First assume no interventions to counteract the EVD outbreak. Susceptibles (*S*) become infected by contacts with infected individuals. Infected first enter the latent phase (*E*), during which they are neither symptomatic nor contagious. This period lasts on average *D*_*E*_ days. The next phase is the prodromal (*P*) phase, which lasts on average *D*_*P*_ days. Prodromals are already infectious, however, not to the full extent, and might develop early symptoms. This phase is followed by the fully infectious phase, during which the disease becomes fully symptomatic. At the end of the prodromal phase, it is determined which percentage of cases will be hospitalized. A fraction *f*_Hosp_ of fully infectious individuals will be hospitalized (*I*_Hosp_), whereas the remaining fraction (*f*_Home_ = 1−*f*_Hosp_) remains at home (*I*_Home_). Medical treatment is assumed to affect the duration of the fully infectious period. It is assumed that this period lasts an average duration of $D_{I_{\text{Hosp}}}$ days in hospital, and $D_{I_{\text{Home}}}$ days at home. At the end of the fully infectious period, individuals either recover or die. The fraction of lethal infections at home ($f_{\text{Dead}}^{\left(\text{Home}\right)}$) is assumed to be larger than among hospitalized cases ($f_{\text{Dead}}^{\left(\text{Hosp}\right)}$). Without proper diagnosis, corpses receive regular funerals (*F*). Importantly, corpses are highly contagious and EVD can spread until the deceased are buried (*B*_*F*_). It takes on average *D*_*F*_ days from death to being buried.

### Adjusting the variance of transition times

An inherent problem with SEIR models are the exponentially distributed transition times from one compartment to the next. Hence, e.g., if the average duration of the latent phase is *D*_*E*_, its variance is $D_{E}^{2}$. To reduce the variance, instead of modeling the early, prodromal, and fully infectious phases each by a single compartment, they are modeled by several equivalent sub-stages (Erlang-stages), through which infected progress successively (see S1 Appendix for details). Let the number of sub-stages in the respective compartments be denoted by *n*_*E*_, *n*_*P*_, $n_{I_{\text{Home}}}$, and $n_{I_{\text{Hosp}}}$. The average durations in the respective sub-stages are *D*_*E*_/*n*_*E*_, *D*_*P*_/*n*_*P*_, $D_{I_{\text{Hosp}}}/n_{I_{\text{Hosp}}}$, and $D_{I_{\text{Home}}}/n_{I_{\text{Home}}}$. As a consequence, the average duration of the latent, prodromal, and fully infectious phase do not change (*D*_*E*_, *D*_*P*_, $D_{I_{\text{Hosp}}}$, and $D_{I_{\text{Home}}}$). However, the durations are now Erlang distributed and their variances become $D_{E}^{2}/n_{E}$, $D_{P}^{2}/n_{P}$, $D_{I_{\text{Hosp}}}^{2}/n_{I_{\text{Hosp}}}$, and $D_{I_{\text{Home}}}^{2}/n_{I_{\text{Home}}}$. Hence, the variance of the duration can be adjusted by the number of Erlang stages (cf. e.g. [71–74]).

### Onset of interventions

To counteract the spread of EVD after its first occurrence at time *t*_Iso_ case isolation, safe funeral practices, and contact tracing are established. To account for these interventions the base model needs to be extended.

### Case isolation

To accommodate case isolation a new compartment for fully infectious but isolated (*I*_Iso_) is introduced, sub-divided into $n_{I_{\text{Iso}}}$ equivalent Erlang stages. It is assumed that, due to precautions, isolated individuals cannot transmit EVD. Since isolated infections are diagnosed, and receive specific medical treatment, the duration during the fully infectious period in isolation is $D_{I_{\text{Iso}}}$ (and $D_{I_{\text{Iso}}}/n_{I_{\text{Iso}}}$ in each corresponding Erlang stage). Also, the fraction of lethal infections in isolation $f_{\text{Dead}}^{\left(\text{Iso}\right)}$ is assumed to be lower than that among hospitalized (and not properly diagnosed) infections.

At the end of the prodromal phase, the proportion of cases *f*_Iso_ which will be isolated is determined. Consequently, the proportions of cases, which will be hospitalized (*f*_Hosp_) and remain at home (*f*_Home_), are adjusted at time *t*_Iso_ to guarantee *f*_Iso_ + *f*_Hosp_ + *f*_Home_ = 1.

### Safe funerals

A characteristic of EVD is that deceased individuals are highly contagious. A recognized concern is the spread of the virus during funeral ceremonies (*F*) [16]. Therefore, all isolated individuals will be buried safely in case of death. To accommodate this, a new compartment of safely buried corpses (*B*_Iso_) is introduced. Transmission does not occur after death if individuals get buried safely. EVD might be properly diagnosed upon death of an individual at home or in hospital, in which case they also receive a safe funeral. It is assumed that fractions *d*_Home_ and *d*_Hosp_ of individuals that died at home or in hospital receive a safe funeral.

### Contact tracing

Contact tracing is a standard practice in many health care systems to contain epidemics. Particularly for diseases as virulent as EVD contact tracing is a cornerstone of disease control. Contact tracing cannot be modeled exactly in an SEIR-type model, since infections are not accounted for on an individual basis. Hence, contact tracing is modeled only approximately.

For contact tracing, infections have to be distinguished into those that are never traced back and not isolated and those that will get isolated and traced back. Contact tracing is not instantaneous, but back-tracking becomes effective after an average duration *D*_*T*_. An individual might be successfully identified by back-tracking during any phase of the disease. To adequately capture this in the model, new compartments for infections (in any of the phases), which will become traced back after an average duration *D*_*T*_ have to be introduced, these are denoted by E*, P*, *I*^(^*^, Home)^, *I*^(^*^, Hosp)^. All infections, subsumed by these newly introduced compartments, will be isolated before recovery or death. This requires the introduction of new compartments for infections which are isolated after being traced back and isolated ($\tilde{E}$, $\tilde{P}$; cf. Fig 1). In the fully infectious phase, we no longer have to distinguish between isolated cases which were found by contact tracing and those diagnosed for other reasons (*I*_Iso_). All newly introduced compartments are again modeled by sub-stages. Since the course of the disease is not affected by whether an infection will be diagnosed in the future, the number of sub-stages and the rates of disease progression in the respective phases do not change, i.e., E* and $\tilde{E}$ are split into *n*_*E*_, P* and $\tilde{P}$ into *n*_*P*_, *I*^(^*^, Hosp)^ into $n_{I_{\text{Hosp}}}$, and *I*^(^*^, Home)^ into $n_{I_{\text{Home}}}$ Erlang stages.

### Limited capacity of isolation wards

Infections which are properly diagnosed will be in isolation during the fully infectious phase. Additionally, infections, which were successfully traced back, will be isolated during any phase of the infection. Isolated infections are treated in quarantine wards and do no longer contribute to disease transmission. However, it assumed that quarantine wards have a maximum capacity *Q*_max_. The number of isolated cases, in excess of this capacity, can no longer be perfectly isolated. It is assumed that only a fraction *p*_Excess_ of these infections is prevented compared to hospital conditions. However, in case an infection that can no longer be properly isolated is lethal, a safe funeral will take place.

### Limited capacity of contact tracing

Due to a lack of capacities, not every individual that should be traced back, can be traced back. There is a maximum capacity *C*_max_ of individuals, whose contacts can be traced back.

### Contact rates

Susceptibles encounter infected individuals (not in isolation) randomly. The relative contagiousness of prodromal individuals, fully infectious individuals at home, fully infectious individuals in hospital, and deceased individuals at unsafe funerals (who are the most contagious) are *c*_*P*_, *c*_*I*_, $c_{I_{\text{Hosp}}}$, and *c*_*F*_, respectively (typically $c_{P}\leq c_{I_{\text{Hosp}}}\leq c_{I}\leq c_{F}$). Individuals that will never get traced back and those who will get traced back are equally contagious in the respective disease phases before they get isolated. These parameters affect the contact rates, in the prodromal, fully infectious, and deceased phases, which are denoted by *β*_*P*_, $\beta_{I_{\text{Home}}}$, $\beta_{I_{\text{Hosp}}}$, and *β*_*F*_, respectively.

### Implementation of the model

The model as described in S1 Appendix was numerically solved by a 4th order Runge-Kutta method. The code was implemented in Python 3.8 using the function solve_ivp as part of the library Scipy, and library Numpy. Graphical output was created using the library Matplotlib. The implementation of the model can be found at GitHub (https://github.com/Maths-against-Malaria/Ebola).

## Results

The model predictions are first described for a baseline scenario. Subsequently, the effect of (i) case isolation, (ii) safe funeral practices, (iii) contact tracing, and (iv) combined measures and their onset on the peak number of infections and mortality are described. The investigated scenarios differ in their feasibility in terms of logistics, equipment, and human resources. The assumptions range from realistic to ideal.

Importantly, case fatality of EVD varies substantially [18], depending on the viral species. In the main text, we describe the situation in which mortality is “moderate” and corresponds to that observed in the 2022 outbreak of the SUDV in Uganda. Additional simulation results assuming high mortality are presented in the supplementary figures for comparison. The interpretation is similar to that in the main text.

Model parameters are adjusted to roughly reflect the situation in a rural area (with poor medical equipment) in Africa with a moderate population of *N* = 10, 000. The choices of model parameters are summarized in S2–S3 Tables. Initially, (*t* = 0) 10 infections are assumed.

### Baseline scenario

**Fig 2:**
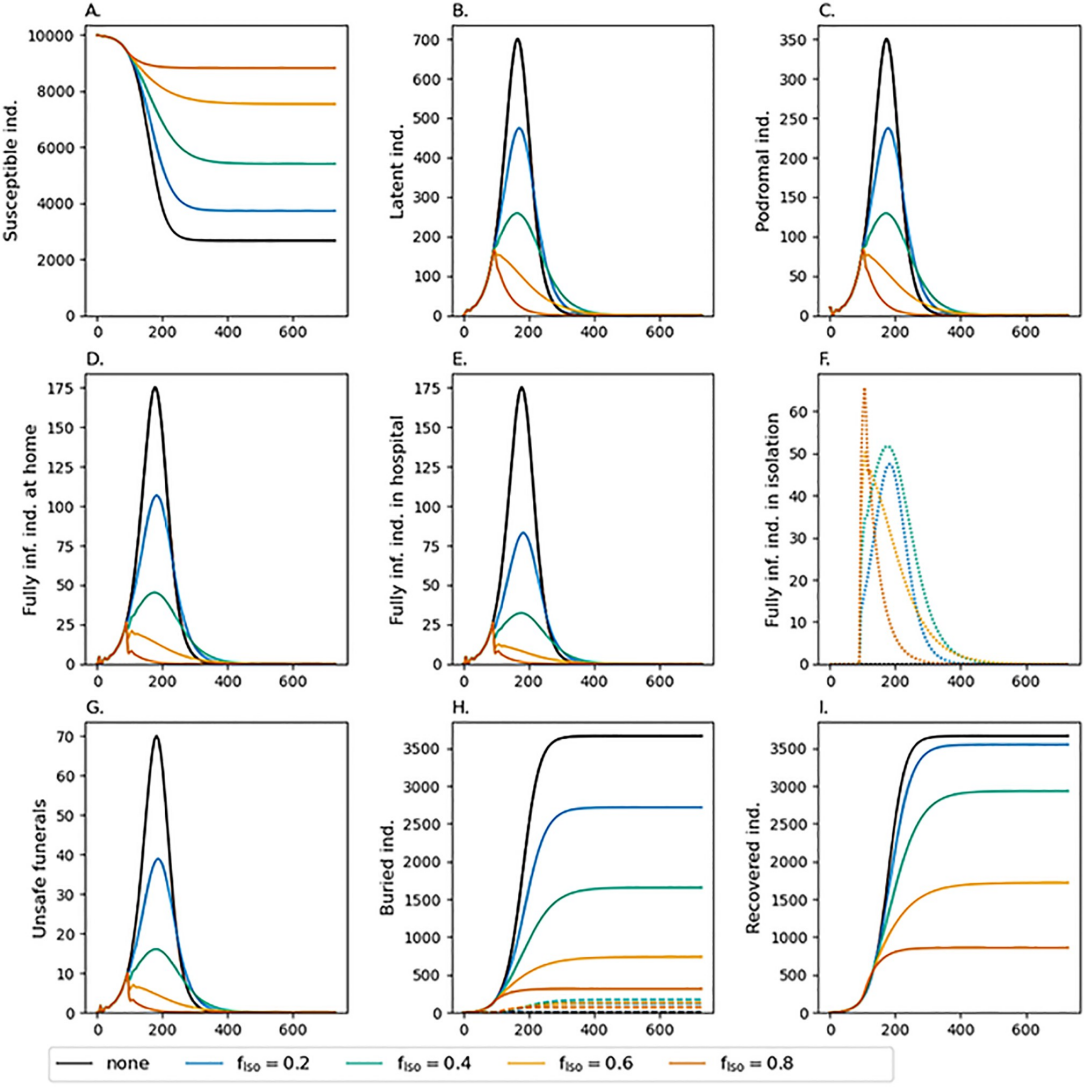
Fraction of isolated *f*_Iso_ infections. Shown are the dynamics of the model for different choices of *f*_Iso_ (colors). Different panels show the number of susceptibles, infected in various phases, unsafe funerals, buried individuals, and recoveries. In the different phases of the infections, the numbers are accumulated over the respective Erlang stages. Isolated infections are shown as dotted lines. In panel (H) the dashed lines show those who have received a safe funeral. Isolation starts at time *t*_Iso_ = 90 days. Moderate mortality is assumed (see S7 Table). All other model parameters used for the simulations are listed in Tables S1–S6 Tables.

In the absence of interventions, the dynamics follow a standard SEIR model, where an epidemic peak occurs after roughly 200 days with 1,354 active infections (see S7 Table and Fig 2, black line). At this point roughly 44% of the population died or recovered, which coincides closely to the classical “herd-immunity” threshold in SIR models of 1−1/*R*_0_ = 0.44 (see Eq. 3.21 in [75]). At this point the epidemic declines and the disease starts to vanish. After roughly 365 days the epidemic is over. The number of remaining susceptibles in the population (2,671) closely resembles the predicted value of 2,675.7 from the final size equation of the standard SIR model (Eq. 1.13 in [75]). The peak number of infections in the latent stage are approximately 700, which are twice those in the prodromal stage (which is intuitive since the latent phase lasts approximately twice as long as the prodromal phase). Roughly 170 cases in the fully infectious phase are hospitalized and the same number remains unhospitalized (this is again intuitive since it is assumed that 50% of cases will be hospitalized). No infection is isolated (Fig 2F) in the baseline scenario and no corpse is buried safely (Fig 2H). More than half of the population dies from the EVD outbreak.

### Case isolation

The effect of case isolation starting at time point *t*_Iso_ = 90 is depicted in Fig 2. Isolating 20% of infections in quarantine wards reduces the slope of new infections. As a consequence, the resulting epidemic peak is lower and occurs slightly later. Hence, also the epidemic ends at a later time point. The overall number of infections is still high with 60% of the population being infected. However, due to isolation, the fraction of infected that remain at home is reduced. Because of better medical treatment in isolation, mortality declines (hence the number of recovered increases in comparison to the baseline scenario) and the number of unsafe funerals decrease by more than 50%. Isolating a larger fraction of infections (40%) has the same qualitative but stronger quantitative effects. Particularly, the epidemic lasts longer, but less than 50% of the population will get infected.

A qualitative change in the dynamics occurs if 60% of cases are isolated. This intervention has an immediate effect. Namely, it readily stops the outbreak with the number of cases starting to decline instantaneously. However, it still takes longer for the epidemic to fade out than in the baseline scenario and overall more than 20% of the population will become infected. Approximately 10% of the population dies from EVD.

The duration of the epidemic is comparable with the baseline scenario if 80% of infections are isolated. In this case around 1,034 individuals become infected and 512 individuals die (S7 Table).

### Safe funerals

**Fig 3:**
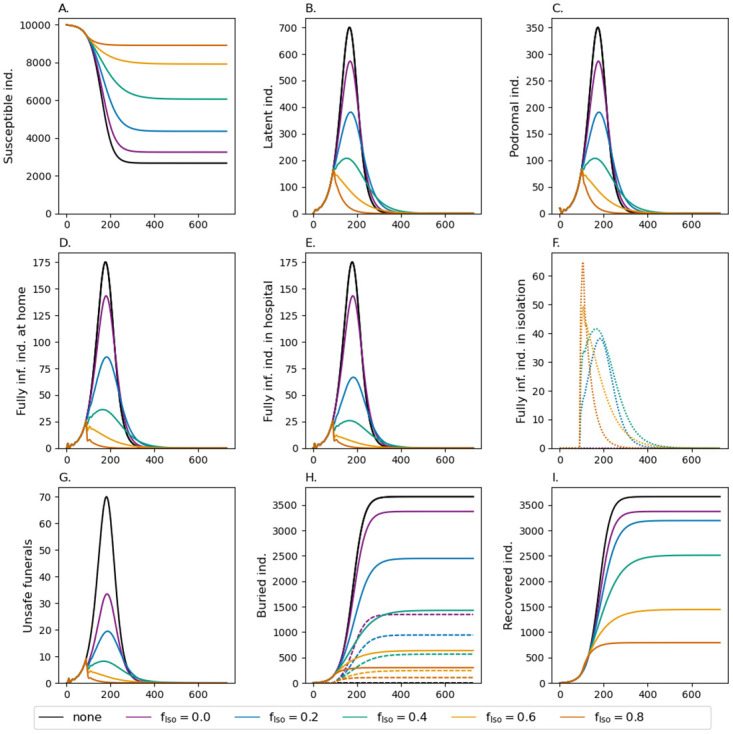
Safe funeral practices. See Fig 2 but combined with safe funeral practices. The fraction of safe funerals after death at home and in hospital are assumed to be *d*_Home_ = 0.16 and *d*_Hosp_ = 0.8, respectively.

Safe funeral practices also help to avoid numerous infectious contacts, especially because corpses are highly contagious. Safe funeral practices without case isolation (purple line in Fig 3), has approximately the same effect on the epidemic as isolating 20% of the infections (blue line in Fig 2). In combination, safe funeral practices amplify the effect of case isolation. Isolating 20% of the population in combination with safe funerals is comparable to isolating 40% of infections without safe funerals, but mortality is higher. The reason is that case isolation leads to better treatment and hence higher chances of survival. The effect of safe funeral practices in combination with case isolation vanishes if larger fractions of cases are isolated. Namely, isolated cases are always buried safely, and the relative number of deceased that additionally receive a safe funeral decreases. Altogether, this renders isolation and safe funeral practices as insufficient to fully control the epidemic.

### Contact tracing

Tracing the contacts of 80% of the cases that enter isolation can help to identify secondary cases and increase the fraction of infections in isolation (S3 Fig). The effects are strongest if isolation by diagnosis (*f*_Iso_) is at 20% to 40%, because many cases will be found and isolated by contact tracing, which would otherwise remain undetected. If initially 80% of infections are diagnosed and isolated, additional isolation by contact tracing has only a small effect. The effects of contact tracing on the epidemic peak and the number of infections are not very strong. However, better treatment in isolation clearly increases the chances of survival. In all scenarios the number of cases in isolation at a time is limited to about 60, which determines the required (effective) quarantine capacity *Q*_max_ (note that in practice also uninfected individuals will get isolated, so the quarantine capacity must be appropriately higher).

#### Extent of contact tracing

**Fig 4:**
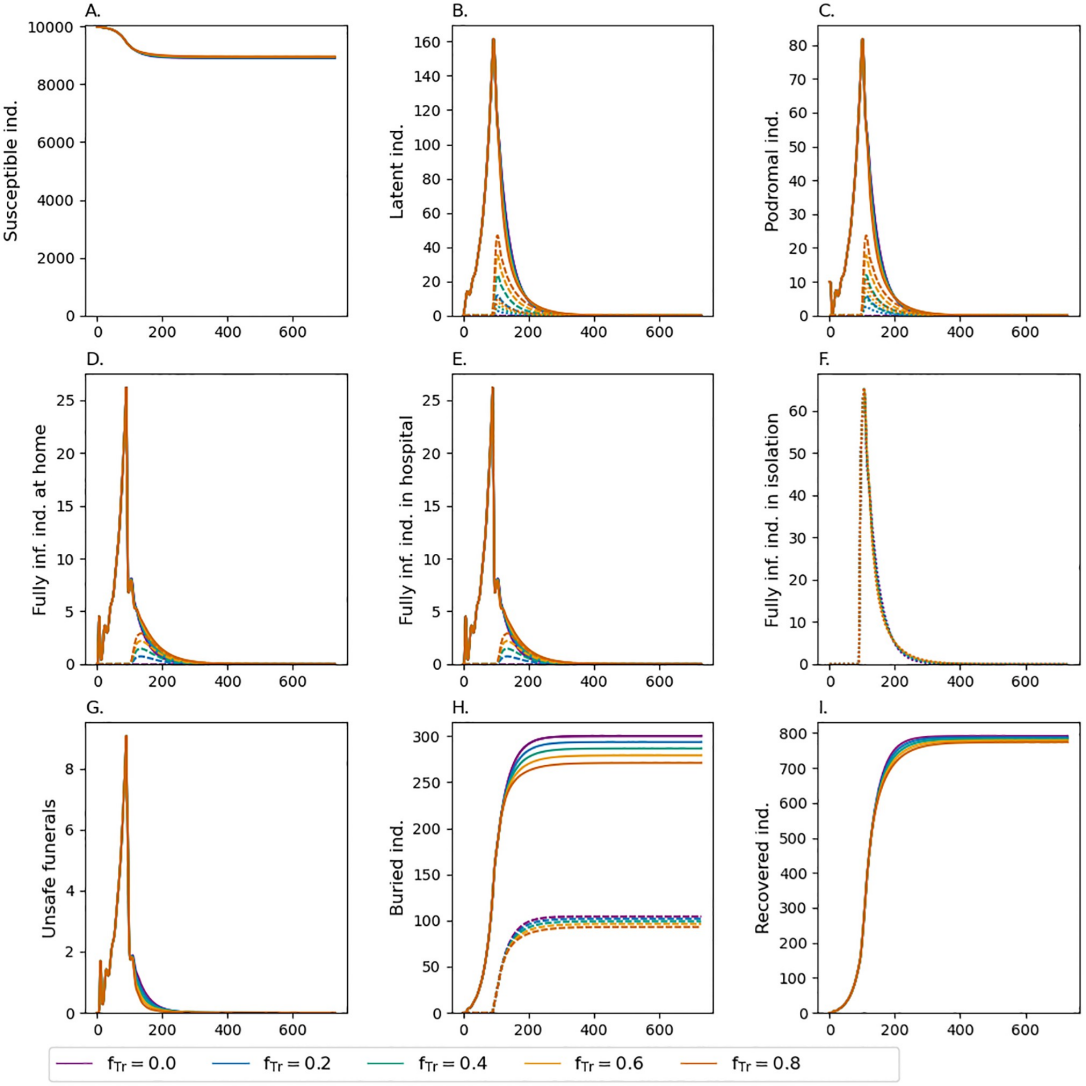
Extent of back-tracking. Effect of fraction of infected individuals who will be traced back ***f*_Tr_**, when the fraction of infections that are isolated is *f*_Iso_ = 0.8 with additional safe funeral practices for lethal cases that occurred outside isolation (*d*_Home_ = 0.16 and *d*_Hosp_ = 0.8). In panels (B-F) the dashed lines show the number of infections that will be traced back at some time in the future (not yet isolated) or are currently traced back (and in isolation). The dotted lines show all individuals currently in isolation. In panel (H) the dashed lines show the numbers of safe funerals that were conducted.

The effect of the extend of contact tracing is illustrated in Fig 4 for the ideal case that 80% of infections are isolated (*f*_Iso_) anyway. Tracing 80% of the contacts of isolated individuals only reduced the number of deaths by about 10%.

#### Efficiency of back tracking

The efficiency of contact tracing can be measured by the average time necessary to isolate suspected infections, i.e., by *D*_*T*_. The effect of the duration necessary to trace back contacts is illustrated in S7 Fig. Since it is assumed that cases become fully infectious on average after 15 days, and they recover or die on average 5 days later, shortening the trace-back time from 20 to 5 days, has a substantial effect. This is especially true when reducing the trace-back time from 25 to 10 days. Any further reduction leads only to marginal improvements. The reason is that 20 days are too long to prevent tertiary cases.

### Combining isolation, contact tracing, and safe funeral practices

**Fig 5:**
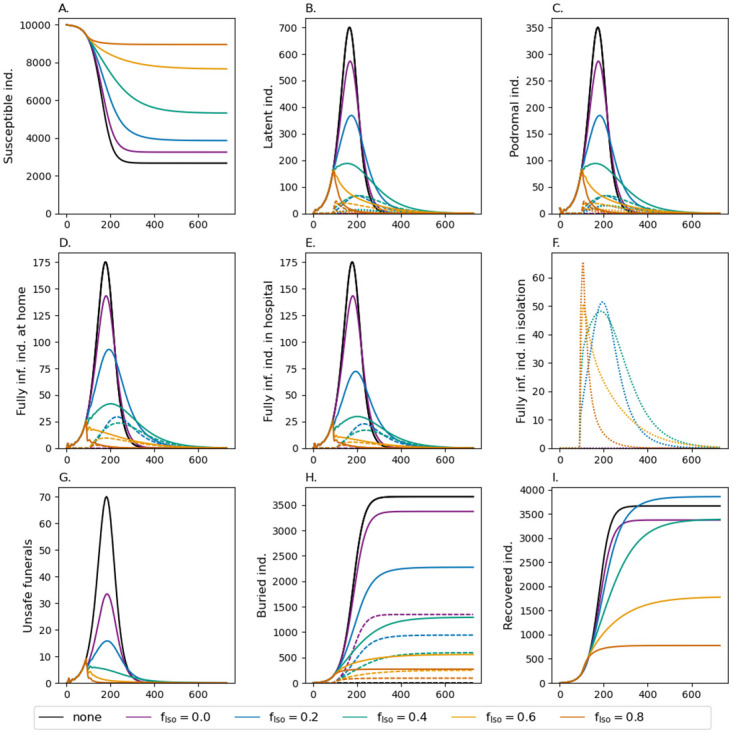
Combination of interventions. See S3 Fig but with additional safe funeral practices for lethal cases that occurred outside isolation (*d*_Home_ = 0.16 and *d*_Hosp_ = 0.8). The average trace-back time is *D*_*T*_ = 21 days. A fraction *f*_Tr_ = 0.8 of contacts of infections in isolation are subject to back-tracking.

As expected, a combining of all three measures leads to the best outcome (Fig 5). The combination of case isolation, safe funeral practices, and back-tracking has a clear effect. Although the effects overlap, e.g., of case isolation and back-tracking, in combination the measures have a synergistic effect. In total, all measures combined compared to just isolation reduces the number of infections and deaths to about 900 and 450, respectively.

#### Onset of interventions

The onset of interventions after the first EVD cases started is very important to curtail the spread of the disease (S10 Fig, S7 Table col. 5,6). Starting to isolate 80% of cases (*f*_Iso_ = 0.8) and back-tracking 80% of their contacts (*f*_Tr_ = 0.8) *t*_Iso_ = 90 after the occurrence of the index cases in combination with safe funeral practices has already a profound effect in containing the epidemic. However, the earlier these measures are implemented the stronger the effect on the epidemic outbreak. In fact, implementing the measures *t*_Iso_ = 30 days after the index cases occurs, contributes to contain the disease outbreak. In fact, the number of infections is reduced from approximately 900 to 120 and the number of deaths from 450 to 55, respectively.

## Discussion

The importance of contact tracing in epidemic management is evident in the current outbreak of the Ebolavirus disease (EVD) due to the Sudan ebolavirus (SUDV) in Uganda [76]. Fast and reliable diagnostics, case isolation, safe funeral practices, and contact tracing along with quarantine are the means of containing outbreaks. This also requires a trained force of healthcare workers and appropriate PPE equipment, potentially on a larger scale [76].

While the appropriateness of the above measures to contain an EVD outbreak is unquestionable, their relative effectiveness in comparison to each other and in combination is ideally quantified, to achieve the optimal response to the outbreak. Given limited resources in terms of qualified personnel, infrastructure, etc., particularly at the onset of an epidemic, one should prioritize the most effective measures.

To quantify the effectiveness of several public health responses, we introduced a deterministic, SEIR-type predictive model, which accounts for them. The model captures aspects which reflect the public health infrastructure of affected areas in terms of the diagnostics, isolation and back-tracing capacities, etc., which determines if the model parameters.

Case isolation and safe funeral practices alone are insufficient to fully contain the epidemic under plausible parameters. This changes if these measures are combined with contact tracing, which additionally contributes to reduce the number of infections and mortality. Importantly, contact tracing and isolation are not independent. Only the contacts of diagnosed infections can be traced, and these will automatically be isolated. Hence, the better the diagnosis (the higher the fraction of isolated infections), the more efficient is the contact tracing.

The onset of contact tracing and case isolation reflects the preparedness of hospitals to diagnose viral hemorrhagic fevers (VHFs) and readily obtain test results. Symptoms awareness of medical professionals is extremely important to obtain proper diagnostics in a timely manner. Namely, on suspicion of a VHF, which can be caused by, e.g., *Arenaviridae* like the Lassa fever, *Flaviviridae* like Dengue or yellow fever, or *Filoviridae* like the EVD or the MVD, patient specimen have to be handled as potential BSL-4 pathogens, and have to be processed by designated institutions. Considering the fact, that EVD outbreaks have zoonotic origin and occur in remote areas, a poor quality infrastructure can lead to substantial delays in the diagnosis of index cases. In the current EVD outbreak in Uganda awareness raised only after six suspicious deaths in the same region, which were linked to the index case [77]. The index case was transferred from a local clinic to a referral hospital, where blood samples were drawn only after two days. When the diagnostic results arrived two days later, the patient already died [77]. However, public health authorities acted within one day to declare an EVD outbreak and issued an Emergency Plan of Action and a National Response plan within a few days [76, 77]. In fact, the Uganda Redcross Society (URCS) developed an EVD preparedness operation in 2018/2019 in response to previous outbreaks [77]. Anyhow, 4–6 weeks have presumably passed since the occurrence of the first infections and the declaration of the outbreak. Awareness of medical professionals was higher during the MVD outbreak in Ghana, however the laboratory results were received relatively late, after the index case died and was buried unsafely, and another suspected case was misdiagnosed, presumably due to contamination of specimens [2]. Importantly, after a single case in Guinea, the MVD outbreak in Ghana marked the only the second one in West Africa.

The onset of contact tracing is shortened by (i) increased awareness of symptoms associated with VHFs, which is achieved by appropriate training of health care practitioners in rural settings; (ii) identification of potentially infective contacts at the earliest suspicion of a VHF; (iii) an adequate network of laboratories with the capacity to receive and process samples within a short time period, e.g., in less than 24 hours; and (iv) a prepared emergency response plan to establish the infrastructure for contact tracing and isolation wards. An earlier onset of contact tracing can mitigate the disease outbreak during the period in which the spread of the disease is determined by random events.

The extent of contact tracing, particularly its follow-up rate, has an enormous impact on containing an EVD outbreak. It is determined by (i) the quality of the emergency response plan, (ii) the available infrastructure in terms of human resources (i.e., properly trained volunteers, community care workers etc.), and (iii) appropriateness of campaigns to raise awareness of suspicious symptoms.

The efficiency of back tracking is reflected by the time necessary to follow up contacts. The results show that a reduction of this time from 20 to 5 days helps to substantially reduce the size of the epidemic outbreak. The trace-back time can be reduced by (i) skilled community care workers, which are well-connected with community leaders (traditional and religious leaders) as appropriate for the cultural specificities of the affected areas; (ii) the quality of the training of healthcare workers and volunteers to appropriate question suspected patients and their households.

Not surprisingly the extent of case isolation has a substantial effect on the epidemic dynamics. The percentage of infections being isolated is determined by (i) the ability of medical professionals to recognize symptoms of VHFs and their willingness to isolate suspicious patients; (ii) the infrastructure and capacity of BSL-4 certified laboratories to process samples; (iii) the availability of isolation wards and capacity to quickly set up appropriate isolation facilities; (iv) the quality of community communication targeted to increase the propensity of the population to seek professional help upon the occurrence of VHF-like symptoms.

The models assumes that safe funeral practices will be conducted upon death in isolation. In practice, this requires teams, which are trained and equipped to perform safe and dignified burials appropriate for the cultural requirements of the affected regions. If the extend of case isolation low, performing safe burials contribute to further reduce the burden of the epidemic. Standard protocols typically involve the use of PPE and disinfecting the body bags of the corpse [78, 79]. However, safe funeral practices alone are insufficient. Notably, the amount of safe burials in access of those, which deceased in isolation, depends on the willingness to perform such a procedure upon alleged death caused by EVD.

Although the model introduced here can be used to explore the optimal strategy to mitigate EVD outbreaks, it will be necessary to quantify the associated costs, which will be varying substantially between countries, depending on their overall economic and political stability, cultural background, available infrastructure, and experience with previous outbreaks. Clearly, model parameters have to be adjusted to a specific situation. However, in areas with suspected zoonotic reservoirs, model parameters can be adjusted to the particular area (in terms of diagnostic, hospital, and logistic capacities) and the optimal response to different EBOV species.

A downside of the SEIR-based model is that contact tracing can only be captured approximately, because individuals are not modeled explicitly. The logic of the model can be adapted in an individual-based model. However, the deterministic approach allows studying the interaction of control interventions without confounding stochastic factors.

The model assumes that isolation is perpetual until recovery or death, i.e., it is disregarded, that individuals being quarantined leave isolation before the onset of symptoms. This can happen if the incubation period of the EVD is underestimated. In fact, the incubation period was underestimated for the MVD, and in two cases during the outbreak in Ghana the onset of symptoms occurred after the completion of the mandatory quarantine [2]. Given that the incubation period of the EVD was, as most other parameters, mainly estimated for the ZEBOV, it cannot be ruled out that the incubation period of a different EBOV species such as the SUDV actually differs. Importantly, there is definitive evidence of spermatogenic transmission of the MARV [80]. Although, this route of transmission is unclear for EBOV, 12 months of safe sex are recommended after the onset of symptoms [78, 79]. Also, the possibility of spermatogenic infection was not included in the model, however, it should be of limited relevance during larger outbreaks.

Notably, the model is not just applicable to EVD, but also to other pathogens causing VHFs, like the Marburg, the Lassa, or the yellow fever virus. However, the model is deterministic, and thus it is only appropriate for diseases which have a sufficiently high base reproduction number so that enough cases occur to ignore stochastic effects. This is questionable for the MVD, which had relatively small outbreaks compared to EVD outbreaks.

Also note that, the public health response might be rather different for other disease outbreaks. Particularly for outbreaks of the ZEBOV or the yellow fever, against which vaccines exist. The reason is that pre- and post-exposure vaccination will be pillars of epidemic management of such outbreaks, which is not captured by the model. However, the present model serves as a blueprint for further model extensions.

## Supporting information

S1 Appendix Mathematical description. (PDF)

S1 Table Population size and model compartments. (PDF)

S2 Table Summary of model parameters describing disease progression and choices for the simulations. (PDF)

S3 Table Summary of model parameters describing infections and choices for the simulations. (PDF)

S4 Table Summary of possible scenarios once interventions control are set up. (PDF)

S5 Table Summary of possible scenarios of safe funeral at home and hospital. (PDF)

S6 Table Summary of possible scenarios (severe and mild mortality) of dead cases at home, in hospital and in isolation. (PDF)

S7 Table Summary of total infected, maximum infected, and total fatalities. (PDF)

S1 Fig Fraction of isolated *f*_Iso_ infections. Shown are the same measures as for Fig 2 but under the assumption of severe mortality (see S7 Table).
(JPG)

S2 Fig Safe funeral practices. Shown are the same measures as for Fig 3 but under the assumption of severe mortality (see S7 Table).
(JPG)

S3 Fig Contact tracing. See Fig 2 but combined with additional contact tracing. A fraction *f*_Tr_ = 0.8 of the contacts of infections in isolation are traced back and isolated themselves. In panels (B-F) the dashed lines show the number of infections that will be traced back at some time in the future (not yet isolated) or are currently traced back (and in isolation). The dotted lines show all individuals currently in isolation. In panel (H) the dashed lines show the numbers of safe funerals that were conducted.
(PNG)

S4 Fig Contact tracing. Shown are the same measures as for S3 Fig but under the assumption of severe mortality (see S7 Table).
(JPG)

S5 Fig Extent of back-tracking. Shown are the same measures as for Fig 4 but under the assumption of severe mortality (see S7 Table).
(JPG)

S6 Fig Extent of back-tracking. Effect of fraction of infected individuals who will be traced back *f*_Tr_ (colors), when the fraction of infections that are isolated is *f*_Iso_ = 0.8 under the assumption of severe mortality (see S7 Table), but without additional save funeral practices. Line types as in S3 Fig.
(PNG)

S7 Fig Efficiency of contact tracing. Shown is the effect of the average trace-back time *D*_*T*_ (colors), assuming 80% of infections are isolated (*f*_Iso_ = 0.8) and 80% of contacts of isolated persons are traced back (*f*_Tr_ = 0.8). Additional safe funeral practices for lethal cases that occurred outside isolation (*d*_Home_ = 0.16 and *d*_Hosp_ = 0.8) are assumed. Line types as in S3 Fig.
(PNG)

S8 Fig Efficiency of contact tracing. Shown are the same measures as for S7 Fig but under the assumption of severe mortality (see S7 Table).
(JPG)

S9 Fig Combination of interventions. Shown are the same measures as for Fig 5 but under the assumption of severe mortality (see S7 Table).
(JPG)

S10 Fig Onset of interventions: Shown is the effect of the onset of interventions *t*_Iso_ (colors). The fraction of infections that are isolated is *f*_Iso_ = 0.8, 80% (*f*_Tr_ = 0.8) of the contacts of isolated patients are subject to back-tracking, and safe funeral practices are conducted outside isolation (*d*_Home_ = 0.16 and *d*_Hosp_ = 0.8). Line types as in S3 Fig.
(PNG)

S11 Fig Onset of interventions: Shown are the same measures as for S10 Fig but under the assumption of severe mortality (see S7 Table). (JPG)
