# Supplementary material for: Predicting the combined effects of case isolation, safe funeral practices, and contact tracing during Ebola virus disease outbreaks
Source: PLoS One. 2023 Jan 17;18(1):e0276351. doi: 10.1371/journal.pone.0276351 (PMC9844901; doi:10.1371/journal.pone.0276351)
Supplement: S1 Appendix — (PDF) [file pone.0276351.s001.pdf]

Appendix: Mathematical description

We study the hypothetical effect of (i) case isolation, (ii) safe funeral ceremonies, and (iii) contact tracing during EVD epidemics during one infective period on disease mortality by an extended SEIR model (see Fig 1 for an illustration).

Basic model compartments

A population of N individuals is assumed. Without interventions, the compartmental model (Fig 1) follows the time change in the number of individuals being susceptible (S), latently infected (E_{Sum}) consisting of n_E sub-states (E_k, $k = 1, \ldots, n_E$), prodromal (P_{Sum}) consisting of n_P sub-states (P_k, $k = 1, \ldots, n_P$), fully infectious at home ($I_{\mathrm{Sum}}^{(\mathrm{Home})}$) consisting of $n_{I_{\mathrm{Home}}}$ sub-states ($I_{k,\mathrm{Home}}$, $k = 1, \ldots, n_{I_{\mathrm{Home}}}$) or in hospital ($I_{\mathrm{Sum}}^{(\mathrm{Hosp})}$) consisting of $n_{I_{\mathrm{Hosp}}}$ sub-states ($I_{k,\mathrm{Hosp}}$, $k = 1, \ldots, n_{I_{\mathrm{Hosp}}}$) or in isolation ($I_{\mathrm{Sum}}^{(\mathrm{Iso})}$) consisting of $n_{I_{\mathrm{Iso}}}$ sub-states ($I_{k,\mathrm{Iso}}$, $k = 1, \ldots, n_{I_{\mathrm{Iso}}}$), finally being recovered (R), or dead, where individuals first await funeral (F) before being buried (B_F). To model intervention, additional compartments are added as described below.

Durations

The average duration of the latent, and prodromal phases are denoted by D_E and D_P, respectively. The duration of the fully infectious disease can in principle be different for hospitalized and isolated cases due to administered treatments. The durations of the fully infectious period for infections at home, in hospital, and in isolation are, respectively, $D_{I_{\mathrm{Home}}}$, $D_{I_{\mathrm{Hosp}}}$, and $D_{I_{\mathrm{Iso}}}$.

Infected individuals have to first progress through a number of equivalent latent sub-states. Hence, the average duration in each latent sub-state is D_E/n_E, and individuals leave the latent sub-states at rate

$$\varepsilon := \frac{n_E}{D_E}. \tag{1a}$$

As a consequence of modeling sub-states the duration in the total latent phase is no longer exponentially distributed, but Erlang distributed with mean D_E and variance D_E/n_E. The advantage is that D_E and n_E can be chosen such that they fit empirical estimates in terms of mean and variance.

After the latent phase, infected individuals progress through prodromal, and fully infectious sub-states in a similar fashion and the rates are, respectively

$$\gamma := \frac{n_P}{D_P}, \quad \delta_{\mathrm{Home}} := \frac{n_{I_{\mathrm{Home}}}}{D_{I_{\mathrm{Home}}}}, \quad \delta_{\mathrm{Hosp}} := \frac{n_{I_{\mathrm{Hosp}}}}{D_{I_{\mathrm{Hosp}}}}, \quad \text{and} \quad \delta_{\mathrm{Iso}} := \frac{n_{I_{\mathrm{Iso}}}}{D_{\mathrm{Iso}}}. \tag{1b}$$

Course of the disease

After the prodromal stage all infections become fully infectious. In addition, a fraction f_{Iso} gets diagnosed with EVD and isolated. This fraction is assumed to be time dependent, i.e., $f_{\mathrm{Iso}}(t)$, reflects that infected will not immediately get diagnosed and isolated at the beginning of the EVD outbreak. Another fraction $f_{\mathrm{Hosp}}(t)$ becomes hospitalized but remains undiagnosed with EVD. The remaining fraction $f_{\mathrm{Home}}(t) = 1 - f_{\mathrm{Iso}}(t) - f_{\mathrm{Hosp}}(t)$ is neither diagnosed nor hospitalized, i.e., the infected individuals stay at home (without proper medical care). Individuals do not die from EVD during the latent or prodromal phase. However, fractions $f_{\mathrm{Dead}}^{(\mathrm{Iso})}$, $f_{\mathrm{Dead}}^{(\mathrm{Hosp})}$, and $f_{\mathrm{Dead}}^{(\mathrm{Home})}$ of symptomatic infections, respectively isolated, hospitalized, and at home, are lethal. Infections not treated in isolation are more likely to be lethal (particularly $f_{\mathrm{Dead}}^{(\mathrm{Home})} \geq f_{\mathrm{Dead}}^{(\mathrm{Hosp})} \geq f_{\mathrm{Dead}}^{(\mathrm{Iso})}$ holds).

Transmission during funerals

Unlike with other diseases, deceased individuals are highly contagious. In fact, EVD can spread during funeral ceremonies (F), which is a recognized concern [1]. If isolated individuals die, they will be buried safely (B_{Iso}), i.e., transmission does not occur after death. The EVD infection of individuals that die at home or in hospital might be properly recognized after death, in which case they also receive a safe funeral. It is assumed that fractions d_{Home} and d_{Hosp} of individuals that died at home or in hospital receive a safe funeral.

Contact tracing

To model contact tracing, we have to distinguish between infections which are never traced back and not isolated and those that will get isolated and traced back.

Contact tracing can help to diagnose more individuals by investigating contacts of the diagnosed cases. In a compartmental model, contact tracing can only be modeled approximately, because infections are not modeled on an individual basis. Contact tracing is not instantaneous. It takes an average duration

$$D_T := \frac{1}{\alpha} \tag{2}$$

until the tracing back becomes effective. To adequately capture this in a compartmental model, we have to introduce new compartments (denoted E^*, P^*, $I^{(*,\text{Home})}$, $I^{(*,\text{Hosp})}$) for infections which will become traced back after an average duration $1/\alpha$. Furthermore, compartments for infections which are isolated after being traced back ($\tilde{E}$, $\tilde{P}$) need to be added to the model (cf. Fig 1). In the fully infectious phase, we no longer distinguish between isolated cases which where found by contact tracing and those diagnosed for other reasons (I_{Iso}).

The model accounts for sub-states of all phases of infected individuals, i.e., those who (i) are never traced back, (ii) are not yet traced back (transient phase), and (iii) are traced back. The respective compartments of those who will get traced back (transient phase) are denoted by E^*_k ($k = 1, \dots, n_E$), P^*_k ($k = 1, \dots, n_P$), $I_k^{(*,\text{Home})}$ ($k = 1, \dots, n_{I_{\text{Home}}}$), and $I_k^{(*,\text{Hosp})}$ ($k = 1, \dots, n_{I_{\text{Hosp}}}$). The compartment of those which are successfully traced back are denoted by $\tilde{E}_k$ ($k = 1, \dots, n_E$), and $\tilde{P}_k$ ($k = 1, \dots, n_P$). The transition rates in the respective compartment are as for infections that are not traced back (given by Eq. 1a–1b)

Onset of interventions

Importantly, intervention measures (case isolation, safe funeral, and contact tracing) will be established only some time after the occurrence of the initial cases. In other words, there is an initial period of t_{Iso} days, after which all measure are implemented.

Regarding isolation and hospitalization we assume

$$f_{\text{Iso}}(t) = \begin{cases} 0 & \text{for } t < t_{\text{Iso}}, \\ f^*_{\text{Iso}} & \text{for } t \geq t_{\text{Iso}}, \end{cases} \tag{3}$$

and

$$f_{\text{Home}}(t) = \begin{cases} f_{\text{Home}} & \text{for } t < t_{\text{Iso}}, \\ f^*_{\text{Home}} & \text{for } t \geq t_{\text{Iso}}. \end{cases} \tag{4}$$

Cumulative numbers of infections

The total number of latently infected which will not get traced back is

$$E_{\text{Sum}}(t) = \sum_{k=1}^{n_E} E_k(t), \tag{5a}$$

while that of latent infections, which will be traced back successfully is

$$E^*_{\text{Sum}}(t) = \sum_{k=1}^{n_E} E^*_k(t), \tag{5b}$$

and that of the latent infection, which were traced back successfully in the latent stage is

$$\tilde{E}_{\text{Sum}}(t) = \sum_{k=1}^{n_E} \tilde{E}_k(t). \tag{5c}$$

Likewise the number of prodromal infections that will not get traced back successfully, will be traced back successfully, and were traced back are, respectively,

$$P_{\text{Sum}}(t) = \sum_{k=1}^{n_P} P_k(t), \tag{6a}$$

$$P^*_{\text{Sum}}(t) = \sum_{k=1}^{n_P} P^*_k(t), \tag{6b}$$

and

$$\tilde{P}_{\text{Sum}}(t) = \sum_{k=1}^{n_P} \tilde{P}_k(t). \tag{6c}$$

Fully infectious individuals remain at home only if they were not successfully traced back or traced back yet. The respective number of these infections are

$$I^{(\text{Home})}_{\text{Sum}}(t) = \sum_{k=1}^{n_{I_{\text{Home}}}} I_{k,\text{Home}}(t), \tag{7a}$$

and

$$I^{(*,\text{Home})}_{\text{Sum}}(t) = \sum_{k=1}^{n_{I_{\text{Home}}}} I^*_{k,\text{Home}}(t). \tag{7b}$$

Similarly, hospitalized fully infectious individuals are either not subject to successful back-tracing or were not yet traced back. The numbers of these infections are, respectively,

$$I^{(\text{Hosp})}_{\text{Sum}}(t) = \sum_{k=1}^{n_{I_{\text{Hosp}}}} I_{k,\text{Hosp}}(t), \tag{8a}$$

and

$$I^{(*,\text{Hosp})}_{\text{Sum}}(t) = \sum_{k=1}^{n_{I_{\text{Hosp}}}} I^*_{k,\text{Hosp}}(t). \tag{8b}$$

Finally, the number of fully infectious individuals which are in isolation (because they were diagnosed with EVD or successfully traced back) is given by

$$I_{\text{Sum}}^{(\text{Iso})}(t) = \sum_{k=1}^{n_{I_{\text{Iso}}}} I_{k,\text{Iso}}(t). \tag{9}$$

Case Isolation

Case isolation is intertwined with contact tracing. Individuals that are successfully traced back will be isolated during any phase of the disease. All individuals, which are successfully traced back, will be in isolation during the fully infectious phase before they eventually recover or die. Additionally, as described above, a fraction $f_{\text{Iso}}(t)$ (which is time dependent) of fully infectious individuals, not subject to successful back tracing, are EVD-diagnosed and isolated.

Isolation lasts until recovery or death. Isolated patients are treated in quarantine wards and do no longer contribute to transmitting the virus. However, quarantine wards have a maximum capacity $Q_{\max}$. The number of isolated cases in excess of the capacities, can no longer be perfectly isolated, in fact only a fraction p_{Excess} of infectious contacts is prevented compared to hospital conditions. The number of infections subject to isolation is given by

$$Q(t) = \tilde{E}_{\text{Sum}}(t) + \tilde{P}_{\text{Sum}}(t) + I_{\text{Sum}}^{(\text{Iso})}(t). \tag{10}$$

The proportion of individuals subject to isolation, which are perfectly isolated is

$$q(t) = \begin{cases} 1 & \text{if} \quad t \geq t_{\text{Iso}} \quad \text{and} \quad Q(t) \leq Q_{\max}, \\ \dfrac{Q_{\max}}{Q(t)} & \text{if} \quad t \geq t_{\text{Iso}} \quad \text{and} \quad Q(t) > Q_{\max}, \\ 0 & \text{if} \quad t < t_{\text{Iso}}. \end{cases} \tag{11}$$

The remaining proportion $1 - q(t)$ of these individuals are isolated imperfectly, i.e., compared to hospitalized patients their contacts are reduced by a fraction $(1 - p_{\text{Excess}})$. Importantly, if they die, they will be sent to safe funeral.

Eventually not every individual that should be traced back, will be traced back. The number of individuals that are supposed to be traced back is

$$\begin{aligned} C(t) = \int_{\tau-1/\alpha}^{\tau} & \varepsilon \tilde{E}_{n_E} + \gamma f_{\text{Iso}}(t)\big(P_{n_P}(t) + P_{n_P}^{*}(t)\big) \\ & + \alpha\Big(P_{\text{Sum}}^{*}(t) + I_{\text{Sum}}^{(*,\text{Home})}(t) + I_{\text{Sum}}^{(*,\text{Hosp})}(t)\Big)dt \end{aligned} \tag{12}$$

$$\begin{aligned} \sim \frac{1}{\alpha}\Big(& \varepsilon \tilde{E}_{n_E} + \gamma f_{\text{Iso}}(t)\big(P_{n_P}(t) + P_{n_P}^{*}(t)\big) \\ & + \alpha\Big(P_{\text{Sum}}^{*}(t) + I_{\text{Sum}}^{(*,\text{Home})}(t) + I_{\text{Sum}}^{(*,\text{Hosp})}(t)\Big)\Big). \end{aligned} \tag{13}$$

Thus, the probability that an individual that should be traced back, is actually traced back is given by

$$c(t) = \begin{cases} 1 & \text{if} \quad t \geq t_{\text{Iso}} \quad \text{and} \quad C(t) \leq C_{\max}, \\ \dfrac{C_{\max}}{C(t)} & \text{if} \quad t \geq t_{\text{Iso}} \quad \text{and} \quad C(t) > C_{\max}, \\ 0 & \text{if} \quad t < t_{\text{Iso}}, \end{cases} \tag{14}$$

where, $C_{\max}$ is the maximum number of individuals that can be traced back.

Contact rates

Susceptibles encounter infected individuals (not in isolation) randomly. The relative contagiousness of prodromal individuals, fully infectious individuals at home, fully infectious individuals in hospital, and deceased individuals at unsafe funerals (who are the most contagious) are c_P, $c_{I_{\mathrm{Home}}}$, $c_{I_{\mathrm{Hosp}}}$, and c_F, respectively (typically $c_P \leq c_{I_{\mathrm{Hosp}}} \leq c_{I_{\mathrm{Home}}} \leq c_F$). Individuals that will never get traced back and those who will get traced back are equally contagious in the respective disease phases before they get isolated. The contact rates depend on the basic reproduction number R_0 described in detail below. This number is determined in a susceptible population before any disease-control interventions are implemented. Note that the fractions of infections which are hospitalized or isolated change over time. Writing $f_{\mathrm{Hosp}} = f_{\mathrm{Hosp}}(0)$ and $f_{\mathrm{Home}} = f_{\mathrm{Home}}(0)$ the contact rates in the respective infectious phases are

$$\beta_P = \frac{c_P}{c_P D_P + f_{\mathrm{Home}} c_{I_{\mathrm{Home}}} D_{I_{\mathrm{Home}}} + f_{\mathrm{Hosp}} c_{I_{\mathrm{Hosp}}} D_{I_{\mathrm{Hosp}}} + u c_F D_F} R_0, \tag{15a}$$

$$\beta_{I_{\mathrm{Home}}} = \frac{c_I}{c_P D_P + f_{\mathrm{Home}} c_{I_{\mathrm{Home}}} D_{I_{\mathrm{Home}}} + f_{\mathrm{Hosp}} c_{I_{\mathrm{Hosp}}} D_{I_{\mathrm{Hosp}}} + u c_F D_F} R_0, \tag{15b}$$

$$\beta_{I_{\mathrm{Hosp}}} = \frac{c_{I_{\mathrm{Hosp}}}}{c_P D_P + f_{\mathrm{Home}} c_{I_{\mathrm{Home}}} D_{I_{\mathrm{Home}}} + f_{\mathrm{Hosp}} c_{I_{\mathrm{Hosp}}} D_{I_{\mathrm{Hosp}}} + u c_F D_F} R_0, \tag{15c}$$

and

$$\beta_F = \frac{c_F}{c_P D_P + f_{\mathrm{Home}} c_{I_{\mathrm{Home}}} D_{I_{\mathrm{Home}}} + f_{\mathrm{Hosp}} c_{I_{\mathrm{Hosp}}} D_{I_{\mathrm{Hosp}}} + u c_F D_F} R_0, \tag{15d}$$

where

$$u := \Big((1 - d_{\mathrm{Home}}) f_{\mathrm{Dead}}^{(\mathrm{Home})} f_{\mathrm{Home}} + (1 - d_{\mathrm{Hosp}}) f_{\mathrm{Dead}}^{(\mathrm{Hosp})} f_{\mathrm{Hosp}} \Big). \tag{15e}$$

Force of infection

During the prodromal and the fully infectious phase, individuals can transmit the virus, the same is true for corpses before being buried (F). Without intervention, transmission is related to the contact rates of the infected in the respective phases. These are modified by case isolation and safe funeral procedures.

Isolated individuals ($\tilde{P}_{\mathrm{Sum}}$, $I_{\mathrm{Sum}}^{(\mathrm{Iso})}$) cannot transmit the disease until the capacity of the quarantine wards are exhausted. Individuals that cannot be isolated in quarantine wards (a fraction $1 - q(t)$ of $\tilde{P}_{\mathrm{Sum}}$ and $I_{\mathrm{Sum}}^{(\mathrm{Iso})}$) are imperfectly isolated (the infectious contacts reduce by a fraction p_{Excess}).

All other individuals in the infectious phases (P_{Sum}, P_{Sum}^*, $I_{\mathrm{Sum}}^{(\mathrm{Home})}$, $I_{\mathrm{Sum}}^{(*,\mathrm{Home})}$, $I_{\mathrm{Sum}}^{(\mathrm{Hosp})}$, $I_{\mathrm{Sum}}^{(*,\mathrm{Hosp})}$, F) are not affected by case isolation and cause infections according to the respective contact rates.

Therefore, the total force of infection is

$$\begin{aligned} \lambda_{\mathrm{Tot}}(t) = & \beta_P \left(P_{\mathrm{Sum}}(t) + P_{\mathrm{Sum}}^*(t) + (1 - p_{\mathrm{Excess}})(1 - q(t)) \tilde{P}_{\mathrm{Sum}}(t) \right) \\ & + \beta_{I_{\mathrm{Home}}} \left(I_{\mathrm{Sum}}^{(\mathrm{Home})}(t) + I_{\mathrm{Sum}}^{(*,\mathrm{Home})}(t) \right) \\ & + \beta_{I_{\mathrm{Hosp}}} \left(I_{\mathrm{Sum}}^{(\mathrm{Hosp})}(t) + I_{\mathrm{Sum}}^{(*,\mathrm{Hosp})}(t) + (1 - p_{\mathrm{Excess}})(1 - q(t)) I_{\mathrm{Sum}}^{(\mathrm{Iso})}(t) \right) \\ & + \beta_F F. \end{aligned} \tag{16}$$

Newly infected individuals will either be subject to contact tracing, i.e., they will be successfully traced back during their infection, or they will not be successfully traced or traced at all. Depending on the outcome of contact tracing, newly infected individuals are either subsumed by the compartments E_1^* or E_1. Consequently the total force of infection is split into two parts corresponding to the force of infection experienced by susceptible that will be successfully traced back λ^* and by those that will not. Hence,

$$\lambda_{\mathrm{Tot}}(t) = \lambda(t) + \lambda^*(t). \tag{17}$$

Fully infectious individuals not subject to back tracing ($I_{\mathrm{Sum}}^{(\mathrm{Home})}$, $I_{\mathrm{Sum}}^{(\mathrm{Hosp})}$, F) only cause infections which will not be traced back. These infections are attributed to λ.

If an individual is successfully traced back, i.e., it was found based on tracing the original infectious contact, this individual might have caused secondary infections, which will also be subject to back tracing. Importantly, only a fraction f_{Tr} of secondary infection can be successfully traced back. Furthermore, the capacity of back tracing is limited, such that only a fraction $c(t)$ of secondary infections, which could be traced back actually can be traced back. Altogether only a fraction $f_{\mathrm{Tr}}c(t)$ of secondary infections of individuals subject to successful back tracing (P_{Sum}^*, $I_{\mathrm{Sum}}^{(*,\mathrm{Home})}$, $I_{\mathrm{Sum}}^{(*,\mathrm{Hosp})}$) will also be traced back and attributed to λ^*. Infections that cannot be properly isolated because the capacity of quarantine wards are reached ($(1-q(t))\tilde{P}_{\mathrm{Sum}}$, $(1-q(t))I_{\mathrm{Sum}}^{(\mathrm{Iso})}$) deserve particular attention. Again only a fraction $f_{\mathrm{Tr}}c(t)$ of secondary infections caused by them will be subject to back tracing. However, their contacts are limited by an amount $1 - p_{\mathrm{Excess}}$.

Prodromal cases also deserve particular attention. While secondary cases of prodromal infections subject to back tracing (P_{Sum}^*, $\tilde{P}_{\mathrm{Sum}}$) are traced back with probability ($f_{\mathrm{Tr}}c(t)$), back tracing of cases subsumed by P_{Sum} are not so obvious. Namely, a fraction $f_{\mathrm{Iso}}(t)$ of prodromals not subject to back tracing yet, will get diagnosed and isolated later. Consequently, they will be subject to back tracing later. Therefore, a fraction of $(f_{\mathrm{Iso}}(t)f_{\mathrm{Tr}}c(t))$ of the contacts of infections subsumed by P_{Sum} will be successfully traced back and attributed to λ^*.

Consequently, the force of infection experienced by suceptibles that will be subject to successful back tracing is

$$\begin{aligned}\lambda^*(t) = f_{\mathrm{Tr}}c(t)\Bigg(&\beta_P\Big(f_{\mathrm{Iso}}(t)P_{\mathrm{Sum}}(t) + P_{\mathrm{Sum}}^*(t) + (1-p_{\mathrm{Excess}})\big(1-q(t)\big)\tilde{P}_{\mathrm{Sum}}(t)\Big)\\ &+ \beta_{I_{\mathrm{Home}}}I_{\mathrm{Sum}}^{(*,\mathrm{Home})}(t) + \beta_{I_{\mathrm{Hosp}}}I_{\mathrm{Sum}}^{(*,\mathrm{Hosp})}(t)\\ &+ \beta_{I_{\mathrm{Hosp}}}(1-p_{\mathrm{Excess}})\big(1-q(t)\big)I_{\mathrm{Sum}}^{(\mathrm{Iso})}(t)\Bigg).\end{aligned} \tag{18a}$$

while that experienced by those that will not be subject to successful back tracing is

$$\begin{aligned}\lambda(t) =&\beta_P\Bigg(\big(1-f_{\mathrm{Iso}}(t)f_{\mathrm{Tr}}c(t)\big)P_{\mathrm{Sum}}(t)\\ &\qquad+\big(1-f_{\mathrm{Tr}}c(t)\big)\Big(P_{\mathrm{Sum}}^*(t) + (1-p_{\mathrm{Excess}})\big(1-q(t)\big)\tilde{P}_{\mathrm{Sum}}(t)\Big)\Bigg)\\ &+\beta_{I_{\mathrm{Home}}}\Bigg(I_{\mathrm{Sum}}^{(\mathrm{Home})}(t) + (1-f_{\mathrm{Tr}}c(t))I_{\mathrm{Sum}}^{(*,\mathrm{Home})}(t)\Bigg)\\ &+\beta_{I_{\mathrm{Hosp}}}\Bigg(I_{\mathrm{Sum}}^{(\mathrm{Hosp})}(t) + (1-f_{\mathrm{Tr}}c(t))I_{\mathrm{Sum}}^{(*,\mathrm{Hosp})}(t)\Bigg)\\ &+\beta_{I_{\mathrm{Hosp}}}\Bigg((1-p_{\mathrm{Excess}})\big(1-q(t)\big)\big(1-f_{\mathrm{Tr}}c(t)\big)\Bigg)I_{\mathrm{Sum}}^{(\mathrm{Iso})}(t)\\ &+\beta_F F.\end{aligned} \tag{18b}$$

Differential Equations

From the above, the system of differential equations describing the dynamics of the spread of EVD follows. The change in the number of susceptibles is given by

$$\frac{dS(t)}{dt} = -\lambda_{\text{Tot}}(t)\frac{S(t)}{N}. \tag{19}$$

The numbers of infected individuals in the latent sub-stages not subject to successful back tracing change according to

$$\frac{dE_1(t)}{dt} = \lambda(t)\frac{S(t)}{N} - \varepsilon E_1(t), \tag{20a}$$

and

$$\frac{dE_k(t)}{dt} = \varepsilon E_{k-1}(t) - \varepsilon E_k(t) \quad \text{for} \quad 2 \le k \le n_E, \tag{20b}$$

while the dynamics of those of latently infected individuals subject to successful back tracing are

$$\frac{dE_1^*(t)}{dt} = \lambda^*(t)\frac{S(t)}{N} - (\alpha + \varepsilon)E_1^*(t), \tag{20c}$$

and

$$\frac{dE_k^*(t)}{dt} = \varepsilon E_{k-1}^*(t) - (\alpha + \varepsilon)E_k^*(t) \quad \text{for} \quad 2 \le k \le n_E. \tag{20d}$$

The numbers of latently infected individuals, who are traced back and isolated change according to

$$\frac{d\tilde{E}_1(t)}{dt} = \alpha E_1^*(t) - \varepsilon \tilde{E}_1(t), \tag{20e}$$

and

$$\frac{d\tilde{E}_k(t)}{dt} = \varepsilon \tilde{E}_{k-1}(t) + \alpha E_k^*(t) - \varepsilon \tilde{E}_k(t) \quad \text{for} \quad 2 \le k \le n_E. \tag{20f}$$

The numbers of prodromal individuals change according to

$$\frac{dP_1(t)}{dt} = \varepsilon E_{n_E}(t) - \gamma P_1(t), \tag{21a}$$

$$\frac{dP_k(t)}{dt} = \gamma P_{k-1}(t) - \gamma P_k(t) \quad \text{for} \quad 2 \le k \le n_P, \tag{21b}$$

$$\frac{dP_1^*(t)}{dt} = \varepsilon E_{n_E}^* - (\alpha + \gamma)P_1^*(t), \tag{21c}$$

$$\frac{dP_k^*(t)}{dt} = \gamma P_{k-1}^*(t) - (\alpha + \gamma)P_k^*(t) \quad \text{for} \quad 2 \le k \le n_P, \tag{21d}$$

$$\frac{d\tilde{P}_1(t)}{dt} = \varepsilon \tilde{E}_{n_E}(t) + \alpha P_1^*(t) - \gamma \tilde{P}_1(t), \tag{21e}$$

and

$$\frac{d\tilde{P}_k(t)}{dt} = \gamma \tilde{P}_{k-1}(t) + \alpha P_k^*(t) - \gamma \tilde{P}_k(t) \quad \text{for} \quad 2 \le k \le n_P. \tag{21f}$$

The numbers fully infectious individuals at home or in hospital, who will not be traced back, change according to

$$\frac{dI_{1,\text{Home}}(t)}{dt} = \gamma f_{\text{Home}}(t) P_{n_P}(t) - \delta_{\text{Home}} I_{1,\text{Home}}(t), \tag{22a}$$

$$\frac{dI_{k,\text{Home}}(t)}{dt} = \delta_{\text{Home}} I_{k-1,\text{Home}}(t) - \delta_{\text{Home}} I_{k,\text{Home}}(t) \quad \text{for} \quad 2 \leq k \leq n_{I_{\text{Home}}}, \tag{22b}$$

$$\frac{dI_{1,\text{Hosp}}(t)}{dt} = \gamma f_{\text{Hosp}}(t) P_{n_P}(t) - \delta_{\text{Hosp}} I_{1,\text{Hosp}}(t), \tag{23a}$$

$$\frac{dI_{k,\text{Hosp}}(t)}{dt} = \delta_{\text{Hosp}} I_{k-1,\text{Hosp}}(t) - \delta_{\text{Hosp}} I_{k,\text{Hosp}}(t) \quad \text{for} \quad 2 \leq k \leq n_{I_{\text{Hosp}}}. \tag{23b}$$

The numbers fully infectious individuals in isolation change according to

$$\begin{aligned}\frac{dI_{1,\text{Iso}}(t)}{dt} =& \gamma \Big(\tilde{P}_{n_P}(t) + f_{\text{Iso}}(t) \big(P_{n_P}(t) + P^*_{n_P}(t) \big) \Big) \\ &+ \alpha \big(I^*_{1,\text{Home}}(t) + I^*_{1,\text{Hosp}}(t) \big) - \delta_{\text{Iso}} I_{1,\text{Iso}}(t),\end{aligned} \tag{24a}$$

$$\begin{aligned}\frac{dI_{k,\text{Iso}}(t)}{dt} =& \delta_{\text{Iso}} I_{k-1,\text{Iso}}(t) + \alpha \big(I^*_{k,\text{Home}}(t) + I^*_{k,\text{Hosp}}(t) \big) - \delta_{\text{Iso}} I_{k,\text{Iso}}(t) \\ & \text{for} \quad 2 \leq k \leq n_{I_{\text{Iso}}}.\end{aligned} \tag{24b}$$

The fully infectious individuals at home and in hospital who will get traced back change according to

$$\frac{dI^*_{1,\text{Home}}(t)}{dt} = \gamma f_{\text{Home}}(t) P^*_{n_P}(t) - (\alpha + \delta_{\text{Home}}) I^*_{1,\text{Home}}(t), \tag{25a}$$

$$\frac{dI^*_{k,\text{Home}}(t)}{dt} = \delta_{\text{Home}} I^*_{k-1,\text{Home}}(t) - (\alpha + \delta_{\text{Home}}) I^*_{k,\text{Home}}(t) \quad \text{for} \quad 2 \leq k < n_{I_{\text{Home}}}, \tag{25b}$$

$$\frac{dI^*_{k,\text{Home}}(t)}{dt} = \delta_{\text{Home}} I^*_{k-1,\text{Home}}(t) - \alpha I^*_{k,\text{Home}}(t) \quad \text{for} \quad k = n_{I_{\text{Home}}}. \tag{25c}$$

$$\frac{dI^*_{1,\text{Hosp}}(t)}{dt} = \gamma f_{\text{Hosp}}(t) P^*_{n_P}(t) - (\alpha + \delta_{\text{Hosp}}) I^*_{1,\text{Hosp}}(t), \tag{26a}$$

$$\frac{dI^*_{k,\text{Hosp}}(t)}{dt} = \delta_{\text{Hosp}} I^*_{k-1,\text{Hosp}}(t) - (\alpha + \delta_{\text{Hosp}}) I^*_{k,\text{Hosp}}(t) \quad \text{for} \quad 2 \leq k < n_{I_{\text{Hosp}}}, \tag{26b}$$

$$\frac{dI^{(*,\text{Hosp})}_k(t)}{dt} = \delta_{\text{Hosp}} I^*_{k-1,\text{Hosp}}(t) - \alpha I^*_{k,\text{Hosp}}(t) \quad \text{for} \quad k = n_{I_{\text{Hosp}}}. \tag{26c}$$

Finally, the number of recovered individuals changes according to

$$\begin{aligned}\frac{dR(t)}{dt} =& \delta_{\text{Home}} \big(1 - f^{(\text{Home})}_{\text{Dead}} \big) I_{n_{I_{\text{Home}}},\text{Home}}(t) + \delta_{\text{Hosp}} \big(1 - f^{(\text{Hosp})}_{\text{Dead}} \big) I_{n_{I_{\text{Hosp}}},\text{Hosp}}(t) \\ &+ \delta_{\text{Iso}} \big(1 - f^{(\text{Iso})}_{\text{Dead}} \big) I_{n_{I_{\text{Iso}}},\text{Iso}}(t).\end{aligned} \tag{27}$$

The number of deceased cases having an unsafe funeral (during which the corpse are highly contagious) changes according to

$$\begin{aligned}\frac{dF(t)}{dt} =& \delta_{\text{Home}} (1 - d_{\text{Home}}) f^{(\text{Home})}_{\text{Dead}} I_{n_{I_{\text{Home}}},\text{Home}}(t) \\ &+ \delta_{\text{Hosp}} (1 - d_{\text{Hosp}}) f^{(\text{Hosp})}_{\text{Dead}} I_{n_{I_{\text{Hosp}}},\text{Hosp}}(t) - \varphi F(t).\end{aligned} \tag{28}$$

The number of corpse, which are buried after a funeral changes according to

$$\frac{dB_F(t)}{dt} = \varphi F(t), \tag{29}$$

while those subject to safe funerals change according to

$$\begin{aligned}\frac{dB_{\mathrm{Iso}}}{dt} =& \delta_{\mathrm{Iso}} f^{(\mathrm{Iso})}_{\mathrm{Dead}} I_{n_{I_{\mathrm{Iso}}},\mathrm{Iso}}(t) + \delta_{\mathrm{Home}} d_{\mathrm{Home}} f^{(\mathrm{Home})}_{\mathrm{Dead}} I_{n_{I_{\mathrm{Home}}},\mathrm{Home}}(t) \\ &+ \delta_{\mathrm{Hosp}} d_{\mathrm{Hosp}} f^{(\mathrm{Hosp})}_{\mathrm{Dead}} I_{n_{I_{\mathrm{Hosp}}},\mathrm{Hosp}}(t).\end{aligned} \tag{30}$$

Basic reproduction number

For convenience the model was parameterized in a way that the basic reproduction number R_0 enters the model as a parameter. Recall, that R_0 is the number of the secondary infections caused by an average single infected individual in a virgin population without any control interventions.

However, according to [2], R_0 is also the spectral radius (maximum absolute eigenvalues) of the next-generation matrix (NGM). In other words, if G denotes the NGM,

$$R_0 := \rho(G) := \max_i |\lambda_i(G)|. \tag{31}$$

Therefore, in the parameter R_0 has to emerge also from the NGM, which will yield the correct definition of the contact rates.

To derive the NGM, first the reduced system of ODEs, needs to be considered and linearized. The reduced system retains only the equations from the original system, which describe the states of infected individuals that are relevant in the absence of interventions. Let $\boldsymbol{X} := [\boldsymbol{E}, \boldsymbol{P}, \boldsymbol{I}_{\mathrm{Home}}, \boldsymbol{I}_{\mathrm{Hosp}}, F]$ be the vector of all variables in the reduced system, where $\boldsymbol{E}, \boldsymbol{P}, \boldsymbol{I}_{\mathrm{Home}}, \boldsymbol{I}_{\mathrm{Hosp}}$ comprise several states each $\boldsymbol{E} = (E_1, \ldots, E_{n_E})$, $\boldsymbol{P} = (P_1, \ldots, P_{n_P})$, $\boldsymbol{I}_{\mathrm{Home}} = (I_{1,\mathrm{Home}}, \ldots, I_{n_{I_{\mathrm{Home}}},\mathrm{Home}})$, $\boldsymbol{I}_{\mathrm{Hosp}} = (I_{1,\mathrm{Hosp}}, \ldots, I_{n_{I_{\mathrm{Hosp}}},\mathrm{Hosp}})$. (In the following, we use brackets to denote vectors or matrices, whose entries are vectors or matrices.) The reduced system first splits into a vector $\mathcal{F}$ describing disease transmission and a vector $\mathcal{V}$ describing the progression of the disease. The Jacobian of these two vectors needs to be derived at the disease free state $\boldsymbol{X}_0$. The NGM is defined as

$$G := -\left[\frac{\partial \mathcal{F}}{\partial \boldsymbol{X}}\bigg|_{\boldsymbol{X}=\boldsymbol{X}_0}\right]\left[\frac{\partial \mathcal{V}}{\partial \boldsymbol{X}}\bigg|_{\boldsymbol{X}=\boldsymbol{X}_0}\right]^{-1} = -\mathscr{F}\mathscr{V}^{-1}, \tag{32}$$

The vector describing transmission is

$$\mathcal{F}(\boldsymbol{X}) := \begin{pmatrix} \lambda(t)\dfrac{S}{N} \\ 0 \\ \vdots \\ 0 \end{pmatrix} = \begin{pmatrix} \mathcal{F}_{E_1} \\ 0 \\ \vdots \\ 0 \end{pmatrix}, \tag{33}$$

where $\mathcal{F}_{E_1} = \lambda(t)\dfrac{S}{N}$. The definition of the force of infection λ_{Tot} in (16) or λ in (18b)

in the absence of interventions yields

$$\frac{\partial \mathcal{F}_{E_1}}{\partial \boldsymbol{E}} := \mathbf{0}, \tag{34a}$$

$$\frac{\partial \mathcal{F}_{E_1}}{\partial \boldsymbol{P}} := \boldsymbol{\beta}_P, \tag{34b}$$

$$\frac{\partial \mathcal{F}_{E_1}}{\partial \boldsymbol{I}_{\text{Home}}} := \boldsymbol{\beta}_{I_{\text{Home}}}, \tag{34c}$$

$$\frac{\partial \mathcal{F}_{E_1}}{\partial \boldsymbol{I}_{\text{Hosp}}} := \boldsymbol{\beta}_{I_{\text{Hosp}}}, \tag{34d}$$

and

$$\frac{\partial \mathcal{F}_{E_1}}{\partial F} := \beta_F, \tag{34e}$$

where (34a)–(34d) are vectors, containing the partial derivative with regard to the various variables (compartments) and $\mathbf{0} = (0, \ldots, 0)$, $\boldsymbol{\beta}_P = (\beta_P, \ldots, \beta_P)$, $\boldsymbol{\beta}_{I_{\text{Home}}} = (\beta_{I_{\text{Home}}}, \ldots, \beta_{I_{\text{Home}}})$, and $\boldsymbol{\beta}_{I_{\text{Hosp}}} = (\beta_{I_{\text{Hosp}}}, \ldots, \beta_{I_{\text{Hosp}}})$, are vectors of dimensions n_E, n_P, $n_{I_{\text{Home}}}$, and $n_{I_{\text{Hosp}}}$, respectively.

Hence, the Jacobian of $\mathcal{F}$, denoted by $\mathscr{F}$, is

$$\begin{aligned}\mathscr{F}(\boldsymbol{X}_0) := \left.\frac{\partial \mathcal{F}}{\partial \boldsymbol{X}}\right|_{\boldsymbol{X}=\boldsymbol{X}_0} &= \begin{bmatrix} \frac{\partial \mathcal{F}_{E_1}}{\partial \boldsymbol{E}} & \frac{\partial \mathcal{F}_{E_1}}{\partial \boldsymbol{P}} & \frac{\partial \mathcal{F}_{E_1}}{\partial \boldsymbol{I}_{\text{Home}}} & \frac{\partial \mathcal{F}_{E_1}}{\partial \boldsymbol{I}_{\text{Hosp}}} & \frac{\partial \mathcal{F}_{E_1}}{\partial F} \\ \mathbf{0} & \mathbf{0} & \mathbf{0} & \mathbf{0} & 0 \\ \vdots & \vdots & \vdots & \vdots & \vdots \\ \mathbf{0} & \mathbf{0} & \mathbf{0} & \mathbf{0} & 0 \end{bmatrix} \\ &= \begin{bmatrix} \mathbf{0} & \boldsymbol{\beta}_P & \boldsymbol{\beta}_{I_{\text{Home}}} & \boldsymbol{\beta}_{I_{\text{Hosp}}} & \beta_F \\ \mathbf{0} & \mathbf{0} & \mathbf{0} & \mathbf{0} & 0 \\ \vdots & \vdots & \vdots & \vdots & \vdots \\ \mathbf{0} & \mathbf{0} & \mathbf{0} & \mathbf{0} & 0 \end{bmatrix},\end{aligned} \tag{35}$$

where $\mathbf{0}$ denote vectors with all elements being 0 of appropriate dimension.

The vector $\mathcal{F}$ describes transmission, i.e., new infections. The vector $\mathcal{V}$ describes the disease progression between all infected states. For the latent state E_1 according to (20), the component describing the disease progression is

$$\mathcal{V}_{E_1} = -\varepsilon E_1(t).$$

For all other states the entries of $\mathcal{V}$ follow directly from the relevant components of the ODE system, (20a)–(20b), (21a)–(21b), (22a)–(22b), (23a)–(23b), and (28) by ignoring all terms, which correspond to interventions. In particular, one obtains

$$\mathcal{V}(\boldsymbol{X}) = \begin{bmatrix} \mathcal{V}_{\boldsymbol{E}} \\ \mathcal{V}_{\boldsymbol{P}} \\ \mathcal{V}_{\boldsymbol{I}_{\text{Home}}} \\ \mathcal{V}_{\boldsymbol{I}_{\text{Hosp}}} \\ \mathcal{V}_F \end{bmatrix}, \tag{36a}$$

where

$$\mathcal{V}_{\boldsymbol{E}} = \begin{pmatrix} \mathcal{V}_{E_1} \\ \vdots \\ \mathcal{V}_{E_k} \end{pmatrix} = \begin{pmatrix} -\varepsilon E_1(t) \\ \vdots \\ \varepsilon E_{k-1}(t) - \varepsilon E_k(t) \end{pmatrix}, \tag{36b}$$

$$\mathcal{V}_{\boldsymbol{P}} = \begin{pmatrix} \mathcal{V}_{P_1} \\ \vdots \\ \mathcal{V}_{P_k} \end{pmatrix} = \begin{pmatrix} \varepsilon E_{n_E}(t) - \gamma P_1(t) \\ \vdots \\ \gamma P_{k-1}(t) - \gamma P_k(t) \end{pmatrix}, \tag{36c}$$

$$\mathcal{V}_{\boldsymbol{I}_{\text{Home}}} = \begin{pmatrix} \mathcal{V}_{I_{1,\text{Home}}} \\ \vdots \\ \mathcal{V}_{I_{k,\text{Home}}} \end{pmatrix} = \begin{pmatrix} \gamma f_{\text{Home}} P_{n_P}(t) - \delta_{\text{Home}} I_{1,\text{Home}}(t) \\ \vdots \\ \delta_{\text{Home}} I_{k-1,\text{Home}}(t) - \delta_{\text{Home}} I_{k,\text{Home}} \end{pmatrix}, \tag{36d}$$

$$\mathcal{V}_{\boldsymbol{I}_{\text{Hosp}}} = \begin{pmatrix} \mathcal{V}_{I_{1,\text{Hosp}}} \\ \vdots \\ \mathcal{V}_{I_{k,\text{Hosp}}} \end{pmatrix} = \begin{pmatrix} \gamma f_{\text{Hosp}} P_{n_P}(t) - \delta_{\text{Hosp}} I_{1,\text{Hosp}}(t) \\ \vdots \\ \delta_{\text{Hosp}} I_{k-1,\text{Hosp}}(t) - \delta_{\text{Hosp}} I_{k,\text{Hosp}} \end{pmatrix}, \tag{36e}$$

$$\mathcal{V}_F = \left(\delta_{\text{Home}} b I_{n_{I_{\text{Home}}},\text{Home}}(t) + \delta_{\text{Hosp}} a I_{n_{I_{\text{Hosp}}},\text{Hosp}}(t) - \varphi F(t) \right), \tag{36f}$$

with

$$a = (1 - d_{\text{Hosp}}) f_{\text{Dead}}^{(\text{Hosp})}, \tag{36g}$$

and

$$b = (1 - d_{\text{Home}}) f_{\text{Dead}}^{(\text{Home})}. \tag{36h}$$

The Jacobian of $\mathcal{V}$, denoted by $\mathscr{V}(\boldsymbol{X}_0)$ consists of the partial derivatives of $\mathcal{V}$ with respect to all states evaluated at $\boldsymbol{X}_0$. For further computation it is convenient to split the Jacobian into blocks corresponding the states $\boldsymbol{E}$, $\boldsymbol{P}$, $\boldsymbol{I}_{\text{Home}}$, $\boldsymbol{I}_{\text{Hosp}}$, and F.

$$\mathscr{V}(\boldsymbol{X}_0) := \left.\frac{\partial \mathcal{V}}{\partial \boldsymbol{X}}\right|_{\boldsymbol{X}=\boldsymbol{X}_0} = \left[\begin{array}{c|c|c|c|c} \frac{\partial \mathcal{V}_{\boldsymbol{E}}}{\partial \boldsymbol{E}} & \frac{\partial \mathcal{V}_{\boldsymbol{E}}}{\partial \boldsymbol{P}} & \frac{\partial \mathcal{V}_{\boldsymbol{E}}}{\partial \boldsymbol{I}_{\text{Home}}} & \frac{\partial \mathcal{V}_{\boldsymbol{E}}}{\partial \boldsymbol{I}_{\text{Hosp}}} & \frac{\partial \mathcal{V}_{\boldsymbol{E}}}{\partial F} \\ \hline \frac{\partial \mathcal{V}_{\boldsymbol{P}}}{\partial \boldsymbol{E}} & \frac{\partial \mathcal{V}_{\boldsymbol{P}}}{\partial \boldsymbol{P}} & \frac{\partial \mathcal{V}_{\boldsymbol{P}}}{\partial \boldsymbol{I}_{\text{Home}}} & \frac{\partial \mathcal{V}_{\boldsymbol{P}}}{\partial \boldsymbol{I}_{\text{Hosp}}} & \frac{\partial \mathcal{V}_{\boldsymbol{P}}}{\partial F} \\ \hline \frac{\partial \mathcal{V}_{\boldsymbol{I}_{\text{Home}}}}{\partial \boldsymbol{E}} & \frac{\partial \mathcal{V}_{\boldsymbol{I}_{\text{Home}}}}{\partial \boldsymbol{P}} & \frac{\partial \mathcal{V}_{\boldsymbol{I}_{\text{Home}}}}{\partial \boldsymbol{I}_{\text{Home}}} & \frac{\partial \mathcal{V}_{\boldsymbol{I}_{\text{Home}}}}{\partial \boldsymbol{I}_{\text{Hosp}}} & \frac{\partial \mathcal{V}_{\boldsymbol{I}_{\text{Home}}}}{\partial F} \\ \hline \frac{\partial \mathcal{V}_{\boldsymbol{I}_{\text{Hosp}}}}{\partial \boldsymbol{E}} & \frac{\partial \mathcal{V}_{\boldsymbol{I}_{\text{Hosp}}}}{\partial \boldsymbol{P}} & \frac{\partial \mathcal{V}_{\boldsymbol{I}_{\text{Hosp}}}}{\partial \boldsymbol{I}_{\text{Home}}} & \frac{\partial \mathcal{V}_{\boldsymbol{I}_{\text{Hosp}}}}{\partial \boldsymbol{I}_{\text{hosp}}} & \frac{\partial \mathcal{V}_{\boldsymbol{I}_{\text{Hosp}}}}{\partial F} \\ \hline \frac{\partial \mathcal{V}_F}{\partial \boldsymbol{E}} & \frac{\partial \mathcal{V}_F}{\partial \boldsymbol{P}} & \frac{\partial \mathcal{V}_F}{\partial \boldsymbol{I}_{\text{Home}}} & \frac{\partial \mathcal{V}_F}{\partial \boldsymbol{I}_{\text{Hosp}}} & \frac{\partial \mathcal{V}_F}{\partial F} \end{array}\right]. \tag{37}$$

The inverse of $\mathscr{V}(\boldsymbol{X}_0)$ needs to be calculated. This is conveniently done, by successively applying a block-wise inversion formula. From (37) one recognizes a blockwise structure,

namely define

$$\mathscr{V}(\boldsymbol{X}_0) = \left[\begin{array}{c|c|c|c|c} A_0 & B_0 & & & \\ \hline C_0 & A_1 & B_1 & & \\ \cline{3-5} & C_1 & A_2 & B_2 & \\ \cline{3-5} & & C_2 & A_3 & B_3 \\ \cline{4-5} & & & C_3 & D_3 \end{array}\right], \tag{38}$$

where we define

$$A_0 := \frac{\partial \mathcal{V}_{\boldsymbol{E}}(\boldsymbol{X})}{\partial \boldsymbol{E}} = -\varepsilon \begin{pmatrix} 1 & & & & \\ -1 & 1 & & & \\ 0 & -1 & 1 & & \\ \vdots & \vdots & \ddots & \ddots & \\ 0 & 0 & \dots & -1 & 1 \end{pmatrix}, \tag{39a}$$

$$B_0 := \begin{pmatrix} \frac{\partial \mathcal{V}_{\boldsymbol{E}}(\boldsymbol{X})}{\partial \boldsymbol{P}} & \frac{\partial \mathcal{V}_{\boldsymbol{E}}(\boldsymbol{X})}{\partial \boldsymbol{I}_{\text{Home}}} & \frac{\partial \mathcal{V}_{\boldsymbol{E}}(\boldsymbol{X})}{\partial \boldsymbol{I}_{\text{Hosp}}} & \frac{\partial \mathcal{V}_{\boldsymbol{E}}(\boldsymbol{X})}{\partial F} \end{pmatrix} = \begin{pmatrix} \mathbf{0}\dots\mathbf{0} & \mathbf{0}\dots\mathbf{0} & \mathbf{0}\dots\mathbf{0} & 0 \end{pmatrix}, \tag{39b}$$

and

$$C_0 := \begin{pmatrix} \frac{\partial \mathcal{V}_{\boldsymbol{P}}(\boldsymbol{X})}{\partial \boldsymbol{E}} \\ \frac{\partial \mathcal{V}_{\boldsymbol{I}_{\text{Home}}}(\boldsymbol{X})}{\partial \boldsymbol{E}} \\ \frac{\partial \mathcal{V}_{\boldsymbol{I}_{\text{Hosp}}}(\boldsymbol{X})}{\partial \boldsymbol{E}} \\ \frac{\partial \mathcal{V}_{F}(\boldsymbol{X})}{\partial \boldsymbol{E}} \end{pmatrix} = \begin{pmatrix} \mathbf{0}\dots\mathbf{0} & \varepsilon \\ \mathbf{0}\dots\mathbf{0} & \mathbf{0} \\ \vdots & \vdots \\ \mathbf{0}\dots\mathbf{0} & \mathbf{0} \\ \mathbf{0}\dots\mathbf{0} & \mathbf{0} \end{pmatrix}. \tag{39c}$$

The remaining blocks are defined as

$$A_1 := \frac{\partial \mathcal{V}_{\boldsymbol{P}}(\boldsymbol{X})}{\partial \boldsymbol{P}} = -\gamma \begin{pmatrix} 1 & & & & \\ -1 & 1 & & & \\ 0 & -1 & 1 & & \\ \vdots & \vdots & \ddots & \ddots & \\ 0 & 0 & \dots & -1 & 1 \end{pmatrix}, \tag{39d}$$

$$B_1 := \begin{pmatrix} \frac{\partial \mathcal{V}_{\boldsymbol{P}}(\boldsymbol{X})}{\partial \boldsymbol{I}_{\text{Home}}} & \frac{\partial \mathcal{V}_{\boldsymbol{P}}(\boldsymbol{X})}{\partial \boldsymbol{I}_{\text{Hosp}}} & \frac{\partial \mathcal{V}_{\boldsymbol{P}}(\boldsymbol{X})}{\partial F} \end{pmatrix} = \begin{pmatrix} \mathbf{0}\dots\mathbf{0} & \mathbf{0}\dots\mathbf{0} & 0 \end{pmatrix}, \tag{39e}$$

$$C_1 := \begin{pmatrix} \frac{\partial \mathcal{V}_{\boldsymbol{I}_{\text{Home}}}(\boldsymbol{X})}{\partial \boldsymbol{P}} \\ \frac{\partial \mathcal{V}_{\boldsymbol{I}_{\text{Hosp}}}(\boldsymbol{X})}{\partial \boldsymbol{P}} \\ \frac{\partial \mathcal{V}_{F}(\boldsymbol{X})}{\partial \boldsymbol{P}} \end{pmatrix} = \begin{pmatrix} \mathbf{0}\dots\mathbf{0} & \gamma f_{\text{Home}} \\ \mathbf{0}\dots\mathbf{0} & \mathbf{0} \\ \vdots & \vdots \\ \mathbf{0}\dots\mathbf{0} & \mathbf{0} \\ \mathbf{0}\dots\mathbf{0} & \gamma f_{\text{Hosp}} \\ \mathbf{0}\dots\mathbf{0} & \mathbf{0} \\ \vdots & \vdots \\ \mathbf{0}\dots\mathbf{0} & \mathbf{0} \\ \mathbf{0}\dots\mathbf{0} & \mathbf{0} \end{pmatrix}, \tag{39f}$$

$$A_2 := \frac{\partial \mathcal{V}_{\boldsymbol{I}_{\text{Home}}}(\boldsymbol{X})}{\partial \boldsymbol{I}_{\text{Home}}} = -\delta_{\text{Home}} \begin{pmatrix} 1 & & & & \\ -1 & 1 & & & \\ 0 & -1 & 1 & & \\ \vdots & \vdots & \ddots & \ddots & \\ 0 & 0 & \dots & -1 & 1 \end{pmatrix}, \tag{39g}$$

$$B_2 := \begin{pmatrix} \frac{\partial \mathcal{V}_{\boldsymbol{I}_{\text{Home}}}(\boldsymbol{X})}{\partial \boldsymbol{I}_{\text{Hosp}}} & \frac{\partial \mathcal{V}_{\boldsymbol{I}_{\text{Home}}}(\boldsymbol{X})}{\partial F} \end{pmatrix} = \begin{pmatrix} \mathbf{0}\dots\mathbf{0} & 0 \end{pmatrix}, \tag{39h}$$

$$C_2 := \begin{pmatrix} \frac{\partial \mathcal{V}_{\boldsymbol{I}_{\text{Hosp}}}(\boldsymbol{X})}{\partial \boldsymbol{I}_{\text{Home}}} \\ \frac{\partial \mathcal{V}_{F}(\boldsymbol{X})}{\partial \boldsymbol{I}_{\text{Home}}} \end{pmatrix} = \begin{pmatrix} \mathbf{0}\dots\mathbf{0} & \mathbf{0} \\ \vdots & \vdots \\ \mathbf{0}\dots\mathbf{0} & \mathbf{0} \\ \mathbf{0}\dots\mathbf{0} & \delta_{\text{Home}} b \end{pmatrix}, \tag{39i}$$

$$A_3 := \frac{\partial \mathcal{V}_{\boldsymbol{I}_{\text{Hosp}}}(\boldsymbol{X})}{\partial \boldsymbol{I}_{\text{Hosp}}} = -\delta_{\text{Hosp}} \begin{pmatrix} 1 & & & & \\ -1 & 1 & & & \\ 0 & -1 & 1 & & \\ \vdots & \vdots & \ddots & \ddots & \\ 0 & 0 & \dots & -1 & 1 \end{pmatrix}, \tag{39j}$$

$$B_3 := \begin{pmatrix} \frac{\partial \mathcal{V}_{\boldsymbol{I}_{\text{Hosp}}}(\boldsymbol{X})}{\partial F} \end{pmatrix} = \begin{pmatrix} \mathbf{0} \end{pmatrix}, \tag{39k}$$

$$C_3 := \begin{pmatrix} \frac{\partial \mathcal{V}_{F}(\boldsymbol{X})}{\partial \boldsymbol{I}_{\text{Hosp}}} \end{pmatrix} = \begin{pmatrix} \mathbf{0}\dots\mathbf{0} & \delta_{\text{Hosp}} a \end{pmatrix}, \tag{39l}$$

and

$$D_3 := \begin{pmatrix} \frac{\partial \mathcal{V}_{F}(\boldsymbol{X})}{\partial F} \end{pmatrix} = -\varphi. \tag{39m}$$

To invert $\mathscr{V}$ we successively apply a block-wise inversion matrix four times, to the

following matrices

$$D_2 = \begin{bmatrix} A_3 & B_3 \\ C_3 & D_3 \end{bmatrix}, \tag{40a}$$

$$D_1 = \begin{bmatrix} A_2 & B_2 \\ C_2 & D_2 \end{bmatrix}, \tag{40b}$$

$$D_0 = \begin{bmatrix} A_1 & B_1 \\ C_1 & D_1 \end{bmatrix}, \tag{40c}$$

and

$$\mathscr{V} = \begin{bmatrix} A_0 & B_0 \\ C_0 & D_0 \end{bmatrix}. \tag{40d}$$

First, using block-wise inversion for D_2 yields

$$\begin{aligned} D_2^{-1} &= \begin{bmatrix} A_3 & B_3 \\ C_3 & D_3 \end{bmatrix}^{-1} \\ &= \begin{bmatrix} \left(A_3 - B_3 C_3 D_3^{-1}\right)^{-1} & -A_3^{-1} B_3 \left(D_3 - A_3^{-1} B_3 C_3\right) \\ -D_3^{-1} C_3 \left(A_3 - B_3 C_3 D_3^{-1}\right)^{-1} & \left(D_3 - A_3^{-1} B_3 C_3\right)^{-1} \end{bmatrix}. \end{aligned} \tag{41}$$

Because $B_3 = \mathbf{0}$, (41) simplifies to

$$D_2^{-1} = \begin{bmatrix} A_3 & B_3 \\ C_3 & D_3 \end{bmatrix}^{-1} = \begin{bmatrix} A_3^{-1} & \mathbf{0} \\ -D_3^{-1} C_3 A_3^{-1} & D_3^{-1} \end{bmatrix}. \tag{42}$$

The inverse matrix of A_3 and D_3 that occur in the above equation are straightforwardly calculated to be

$$A_3^{-1} = -\frac{1}{\delta_{\text{Hosp}}} \begin{pmatrix} 1 & & & & \\ 1 & 1 & & & \\ 1 & 1 & 1 & & \\ \vdots & \vdots & \ddots & \ddots & \\ 1 & 1 & \dots & 1 & 1 \end{pmatrix} \tag{43a}$$

and

$$D_3^{-1} = -\frac{1}{\varphi}, \tag{43b}$$

from which D_2^{-1} follows explicitly. Namely,

$$D_2^{-1} = \begin{pmatrix} -\frac{1}{\delta_{\text{Hosp}}} & & & & \\ -\frac{1}{\delta_{\text{Hosp}}} & -\frac{1}{\delta_{\text{Hosp}}} & & & \\ \vdots & \vdots & \ddots & & \\ -\frac{1}{\delta_{\text{Hosp}}} & -\frac{1}{\delta_{\text{Hosp}}} & \dots & -\frac{1}{\delta_{\text{Hosp}}} & \\ -\frac{a}{\varphi} & -\frac{a}{\varphi} & \dots & -\frac{a}{\varphi} & -\frac{1}{\varphi} \end{pmatrix}. \tag{44}$$

Next, block-wise inversion of D_1 yields

$$D_1^{-1} = \begin{bmatrix} A_2 & B_2 \\ C_2 & D_2 \end{bmatrix}^{-1} = \begin{bmatrix} A_2^{-1} & \mathbf{0} \\ -D_2^{-1}C_2A_2^{-1} & D_2^{-1} \end{bmatrix}, \tag{45}$$

where D_2^{-1} is given by (44) and A_2^{-1} is derived similarly as A_3^{-1}, namely

$$A_2^{-1} = -\frac{1}{\delta_{\text{Home}}} \begin{pmatrix} 1 & & & & \\ 1 & 1 & & & \\ 1 & 1 & 1 & & \\ \vdots & \vdots & \ddots & \ddots & \\ 1 & 1 & \dots & 1 & 1 \end{pmatrix}. \tag{46}$$

The product $-D_2^{-1}C_2A_2^{-1}$ is straightforwardly derived.

Similarly, D_0^{-1} is derived as

$$D_0^{-1} = \begin{bmatrix} A_1 & B_1 \\ C_1 & D_1 \end{bmatrix}^{-1} = \begin{bmatrix} A_1^{-1} & \mathbf{0} \\ -D_1^{-1}C_1A_1^{-1} & D_1^{-1} \end{bmatrix}, \tag{47}$$

where A_1^{-1} is derived in the same way as A_2^{-1} or A_3^{-1}, namely

$$A_1^{-1} = -\frac{1}{\gamma} \begin{pmatrix} 1 & & & & \\ 1 & 1 & & & \\ 1 & 1 & 1 & & \\ \vdots & \vdots & \ddots & \ddots & \\ 1 & 1 & \dots & 1 & 1 \end{pmatrix}. \tag{48}$$

The product $-D_1^{-1}C_1A_1^{-1}$ follows immediately.

Finally, the matrix $\mathscr{V}$ is inverted as

$$\mathscr{V}^{-1} = \begin{bmatrix} A_0 & B_0 \\ C_0 & D_0 \end{bmatrix}^{-1} = \begin{bmatrix} A_0^{-1} & \mathbf{0} \\ -D_0^{-1}C_0A_0^{-1} & D_0^{-1} \end{bmatrix}, \tag{49}$$

where A_0^{-1} is derived to be

$$A_0^{-1} = -\frac{1}{\varepsilon} \begin{pmatrix} 1 & & & & \\ 1 & 1 & & & \\ 1 & 1 & 1 & & \\ \vdots & \vdots & \ddots & \ddots & \\ 1 & 1 & \dots & 1 & 1 \end{pmatrix}. \tag{50}$$

Although $-D_0^{-1}C_0A_0^{-1}$ follows straightforwardly, it is not necessary to calculate it explicitly. The reason is that we need only to calculate the eigenvalues of the product

$$G = -\mathscr{F}\mathscr{V}^{-1}. \tag{51}$$

From (35) and (37) it follows that only the first row of G contains non-zero elements. Thus, the only eigenvalue different from zero is the first entry of the matrix G, i.e., the product of the first row of $\mathscr{F}$ and the first column of $\mathscr{V}$. A tedious but simple calculation shows

$$R_0 := \rho(G) = \frac{n_P\beta_P}{\gamma} + \frac{n_{I_{\text{Home}}}\beta_{I_{\text{Home}}}f_{\text{Home}}}{\delta_{\text{Home}}} + \frac{n_{I_{\text{Hosp}}}\beta_{I_{\text{Hosp}}}f_{\text{Hosp}}}{\delta_{\text{Hosp}}} + \frac{\beta_F u}{\varphi}. \tag{52a}$$

Using the definition of γ, δ_{Home}, δ_{Hosp}, and φ (1b) yields

$$R_0 = \beta_P D_P + \beta_{I_{\text{Home}}} f_{\text{Home}} D_{I_{\text{Home}}} + \beta_{I_{\text{Hosp}}} f_{\text{Hosp}} D_{I_{\text{Hosp}}} + \beta_F u D_F. \tag{52b}$$

Computation of contact rates

The contact rates are proportional to the product of R_0 and the relative contagiousness in the respective phase of the disease (c_P, c_I, $c_{I_{\text{Hosp}}}$, and c_F), i.e.,

$$\beta_P = Kc_P R_0, \tag{53a}$$

$$\beta_{I_{\text{Home}}} = Kc_{I_{\text{Home}}} R_0, \tag{53b}$$

$$\beta_{I_{\text{Hosp}}} = Kc_{I_{\text{Hosp}}} R_0, \tag{53c}$$

$$\beta_F = Kc_F R_0. \tag{53d}$$

Deriving the appropriate proportional constant K yields 15a, 15b, 15c, and 15d.

Remember, the basic reproduction number R_0 has to emerge as the spectral radius of the NGM. Combining (53) and (52b), gives

$$\begin{aligned} R_0 &= Kc_P R_0 D_P + f_{\text{Home}} K c_{I_{\text{Home}}} R_0 D_{I_{\text{Home}}} + f_{\text{Hosp}} K c_{I_{\text{Hosp}}} R_0 D_{I_{\text{Hosp}}} + uKc_F R_0 D_F, \\ R_0 &= KR_0\Big(c_P D_P + f_{\text{Home}} c_{I_{\text{Home}}} D_{I_{\text{Home}}} + f_{\text{Hosp}} c_{I_{\text{Hosp}}} D_{I_{\text{Hosp}}} + uc_F D_F\Big), \end{aligned} \tag{54}$$

which implies

$$K = \frac{1}{c_P D_P + f_{\text{Home}} c_{I_{\text{Home}}} D_{I_{\text{Home}}} + f_{\text{Hosp}} c_{I_{\text{Hosp}}} D_{I_{\text{Hosp}}} + uc_F D_F}. \tag{55}$$

Substituting (55) into (53) yields (53a), (53b), (53c) and (53d).

References

1. Victory KR, Coronado F, Ifono SO, Soropogui T, Dahl BA. Ebola transmission linked to a single traditional funeral ceremony—Kissidougou, Guinea, December, 2014–January 2015. MMWR Morbidity and mortality weekly report. 2015;64(14):386.

2. Diekmann O, Heesterbeek JAP, Metz JA. On the definition and the computation of the basic reproduction ratio R 0 in models for infectious diseases in heterogeneous populations. Journal of mathematical biology. 1990;28(4):365–382. doi:10.1007/BF00178324.
