# Supplementary material for: Predicting the combined effects of case isolation, safe funeral practices, and contact tracing during Ebola virus disease outbreaks
Source: PLoS One. 2023 Jan 17;18(1):e0276351. doi: 10.1371/journal.pone.0276351 (PMC9844901; doi:10.1371/journal.pone.0276351)
Supplement: S1 Table — (PDF) [file pone.0276351.s002.pdf]

S1 Table. Population size and model compartments.

Name	Description	Value for $t = 0$
N	Population size	10,000
$S(t)$	No. susceptible	9,990
$E(t)$	No. infected in latent stage ($1 \leq k \leq n_E$)	0
$E^*(t)$	No. latent infected ind. who are going to be traced back ($1 \leq k \leq n_E$)	0
$\tilde{E}(t)$	No. latent infected ind. who are traced back ($1 \leq k \leq n_E$)	0
$P_1(t)$	No. infected ind. in first prodromal Erlang stage	10
$P_k(t)$	No. infected ind. in kth prodromal Erlang stage ($2 \leq k \leq n_P$)	0
$P^*(t)$	No. prodromal infected ind. who will get traced back ($1 \leq k \leq n_P$)	0
$\tilde{P}(t)$	No. prodromal infected ind. who are traced back ($1 \leq k \leq n_P$)	0
$I_{\text{Home}}(t)$	No. ind. in fully infectious stages at home ($1 \leq k \leq n_{I_{\text{Home}}}$)	0
$I_{\text{Hosp}}(t)$	No. ind. in fully infectious stages in hospital ($1 \leq k \leq n_{I_{\text{Hosp}}}$)	0
$I_{\text{Iso}}(t)$	No. ind. in fully infectious stages in isolation ($1 \leq k \leq n_{I_{\text{Iso}}}$)	0
$I^{(*,\text{Home})}(t)$	No. ind. in fully infectious stages at home who will get traced back ($1 \leq k \leq n_{I_{\text{Home}}}$)	0
$I^{(*,\text{Hosp})}(t)$	No. ind. in fully infectious stages in hospital who will get traced back ($1 \leq k \leq n_{I_{\text{Hosp}}}$)	0
$R(t)$	No. recovered individuals	0
$F(t)$	No. individuals having a funeral	0
$B_{\text{F}}(t)$	No. buried who had unsafe funeral	0
$B_{\text{Iso}}(t)$	No. buried who had safe funeral	0
