# Supplementary material for: Predicting the combined effects of case isolation, safe funeral practices, and contact tracing during Ebola virus disease outbreaks
Source: PLoS One. 2023 Jan 17;18(1):e0276351. doi: 10.1371/journal.pone.0276351 (PMC9844901; doi:10.1371/journal.pone.0276351)
Supplement: S2 Table — (PDF) [file pone.0276351.s003.pdf]

S2 Table. Parameters of disease progression.

Name	Description	Value
n_{E}	No. latency Erlang stages	16
n_{P}	No. prodromal Erlang stages	16
$n_{I_{\mathrm{Home}}}$	No. fully infectious (at home) Erlang stages	16
$n_{I_{\mathrm{Hosp}}}$	No. fully infectious (in hospital) Erlang stages	16
$n_{I_{\mathrm{Iso}}}$	No. fully infectious (in isolation) Erlang stages	16
D_E	Average duration of latency period (days)	10
D_P	Average duration of prodromal period (days)	5
$D_{I_{\mathrm{Home}}}$	Average duration of fully infectious period at home (days)	5
$D_{I_{\mathrm{Hosp}}}$	Average duration of fully infectious period in hospital (days)	5
$D_{I_{\mathrm{Iso}}}$	Average duration of fully infectious period in isolation (days)	5
D_F	Average duration from death to being buried (days)	2
D_T	Average trace back time (days)	21
ε	Transition rate of latent states	n_{E}/D_E
γ	Transition rate of prodromal states	n_{P}/D_P
δ_{Home}	Transition rate of fully infectious states	$n_{I_{\mathrm{Home}}}/D_{I_{\mathrm{Home}}}$
δ_{Hosp}	Transition rate of fully infectious states	$n_{I_{\mathrm{Hosp}}}/D_{I_{\mathrm{Hosp}}}$
δ_{Iso}	Transition rate of fully infectious states	$n_{I_{\mathrm{Iso}}}/D_{I_{\mathrm{Iso}}}$
φ	Transition rate of unsafe funeral	$1/D_F$
α	Transition rate of successful tracing back time	$1/D_T$
$f_{\mathrm{Dead}}^{(\mathrm{Home})}$	Fraction of infected ind. in home isolation who die from the disease	[0.6, 0.9]
$f_{\mathrm{Dead}}^{(\mathrm{Hosp})}$	Fraction of infected ind. in hospital who die from the disease	[0.4, 0.6]
$f_{\mathrm{Dead}}^{(\mathrm{Iso})}$	Fraction of infected ind. in isolation (ETU) who die from the disease	[0.1, 0.3]
d_{Hosp}	Fraction of individuals who died in hospital that are buried safely	[0, 0.8]
d_{Home}	Fraction of individuals who died at home that are buried safely	[0, 0.16]
