# Supplementary material for: Predicting the combined effects of case isolation, safe funeral practices, and contact tracing during Ebola virus disease outbreaks
Source: PLoS One. 2023 Jan 17;18(1):e0276351. doi: 10.1371/journal.pone.0276351 (PMC9844901; doi:10.1371/journal.pone.0276351)
Supplement: S3 Table — (PDF) [file pone.0276351.s004.pdf]

S3 Table. Parameters of force of infection and related to countermeasures.

Name	Description	Value/Eq.
$\lambda_{\mathrm{Tot}}(t)$	Total force of infection	$\lambda(t) + \lambda^*(t)$ Eq. (17)
$\lambda(t)$	Force of infection leading to infections without tracing back	Eq. (18a)
$\lambda^*(t)$	Force of infection leading to infections with tracing back and isolation	Eq. (18b)
f_{Tr}	Fraction of isolated ind. who will be traced back	$0 \leq f_{\mathrm{Tr}} \leq 1$
p_{Excess}	Effectiveness of isolation of ind. with Ebola diagnosis in normal hospital	$0 \leq p_{\mathrm{Excess}} \leq 1$
R_0	Basic reproduction number	1.8
c_P	Relative contagiousness in prodromal stage	0.3
$c_{I_{\mathrm{Home}}}$	Relative contagiousness of fully inf. stage at home	0.6
$c_{I_{\mathrm{Hosp}}}$	Relative contagiousness of fully inf. stage at hospital (adjusted by contact reduction)	0.5
c_F	Relative contagiousness after death before being buried unsafely	1
$Q_{\max}$	Maximum capacity of the isolation units	N
$C_{\max}$	Maximum capacity (number of individuals) of tracing back	200
t_{Iso}	Day when measures start	day 90
