# Supplementary material for: Predicting the combined effects of case isolation, safe funeral practices, and contact tracing during Ebola virus disease outbreaks
Source: PLoS One. 2023 Jan 17;18(1):e0276351. doi: 10.1371/journal.pone.0276351 (PMC9844901; doi:10.1371/journal.pone.0276351)
Supplement: S4 Table — (PDF) [file pone.0276351.s005.pdf]

S4 Table. Scenarios of treatments.

Intervention starting	$t \leq t_{\mathrm{Iso}}$	$t \geq t_{\mathrm{Iso}}$			
Scenarios	baseline	scenario 1	scenario 2	scenario 3	scenario 4
f_{Home}	0.5	0.45	0.35	0.25	0.1
f_{Hosp}	0.5	0.35	0.25	0.15	0.1
f_{Iso}	0	0.2	0.4	0.6	0.8
