# Supplementary material for: Predicting the combined effects of case isolation, safe funeral practices, and contact tracing during Ebola virus disease outbreaks
Source: PLoS One. 2023 Jan 17;18(1):e0276351. doi: 10.1371/journal.pone.0276351 (PMC9844901; doi:10.1371/journal.pone.0276351)
Supplement: S5 Table — (PDF) [file pone.0276351.s006.pdf]

S5 Table. Scenarios of safe funeral at home and hospital.

Scenarios	baseline	scenario 1	scenario 2	scenario 3	scenario 4
d_{Home}	0	0.04	0.08	0.12	0.16
d_{Hosp}	0	0.2	0.4	0.6	0.8
