# Supplementary material for: Predicting the combined effects of case isolation, safe funeral practices, and contact tracing during Ebola virus disease outbreaks
Source: PLoS One. 2023 Jan 17;18(1):e0276351. doi: 10.1371/journal.pone.0276351 (PMC9844901; doi:10.1371/journal.pone.0276351)
Supplement: S6 Table — (PDF) [file pone.0276351.s007.pdf]

S6 Table. Scenarios of death rates.

Scenarios	$f_{\text{Dead}}^{(\text{Home})}$	$f_{\text{Dead}}^{(\text{Hosp})}$	$f_{\text{Dead}}^{(\text{Iso})}$
Severe mortality	0.9	0.6	0.3
Moderate mortality	0.6	0.4	0.1
