# Supplementary material for: Predicting the combined effects of case isolation, safe funeral practices, and contact tracing during Ebola virus disease outbreaks
Source: PLoS One. 2023 Jan 17;18(1):e0276351. doi: 10.1371/journal.pone.0276351 (PMC9844901; doi:10.1371/journal.pone.0276351)
Supplement: S7 Table — (PDF) [file pone.0276351.s008.pdf]

S7 Table. Results (under severe mortality).

f_{Iso}	0	0.8	0.8	0.8	0.8	0.8
f_{Tr}	0	0	0.8	0	0.8	0.8
d_{Home}	0	0	0	0.16	0.16	0.16
d_{Hosp}	0	0	0	0.8	0.8	0.8
t_{Iso}	–	90	90	90	90	30
total infected	7329	1034	965	942	903	123
max. infected	1354	270	270	263	263	37
total deaths	5497	512	466	476	443	55
