# Supplementary figures and images for: Predicting the combined effects of case isolation, safe funeral practices, and contact tracing during Ebola virus disease outbreaks
Source: PLoS One. 2023 Jan 17;18(1):e0276351. doi: 10.1371/journal.pone.0276351 (PMC9844901; doi:10.1371/journal.pone.0276351)

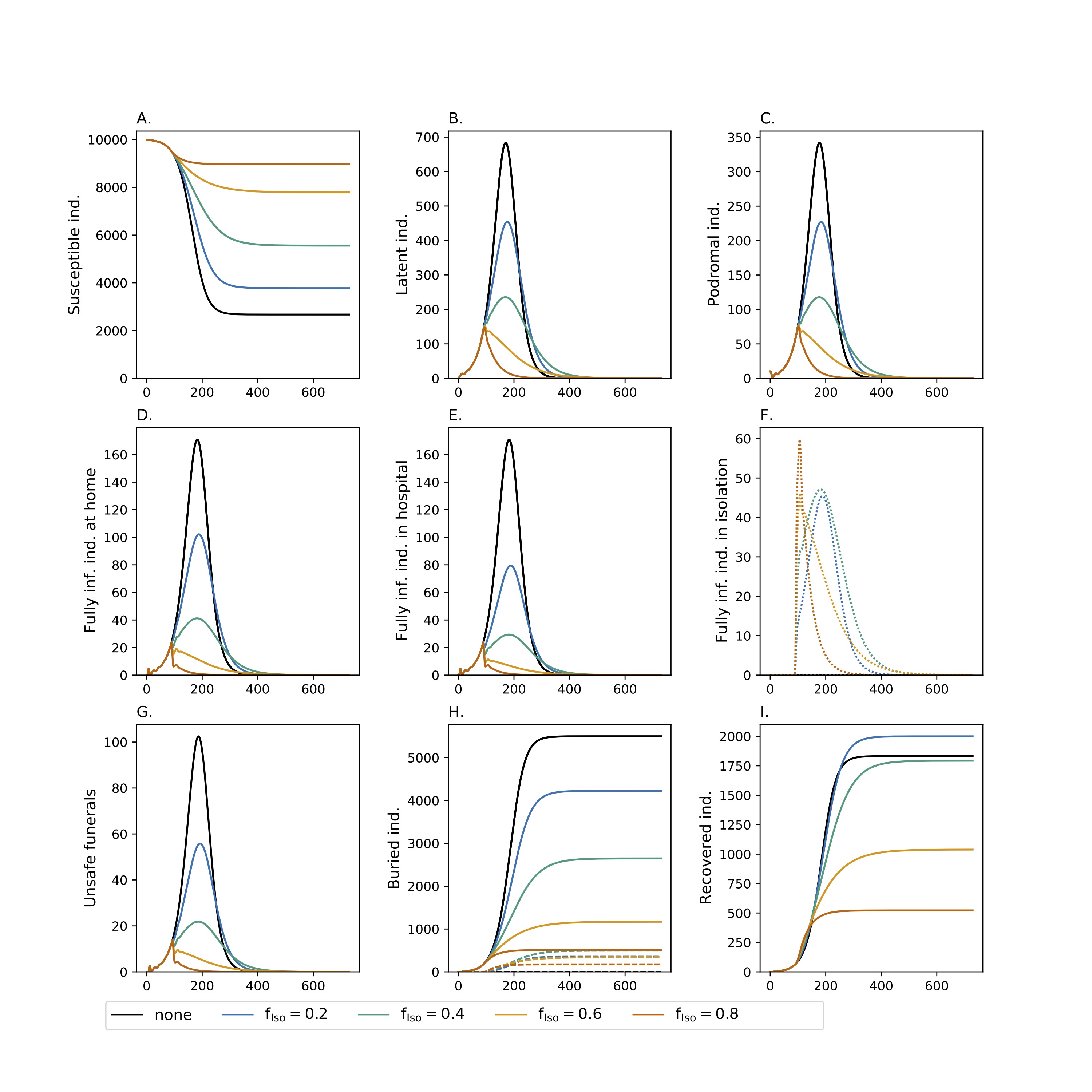

Supplement: S1 Fig — Shown are the same measures as for Fig 2 but under the assumption of severe mortality (see S7 Table). (JPG) [file pone.0276351.s009.jpg]

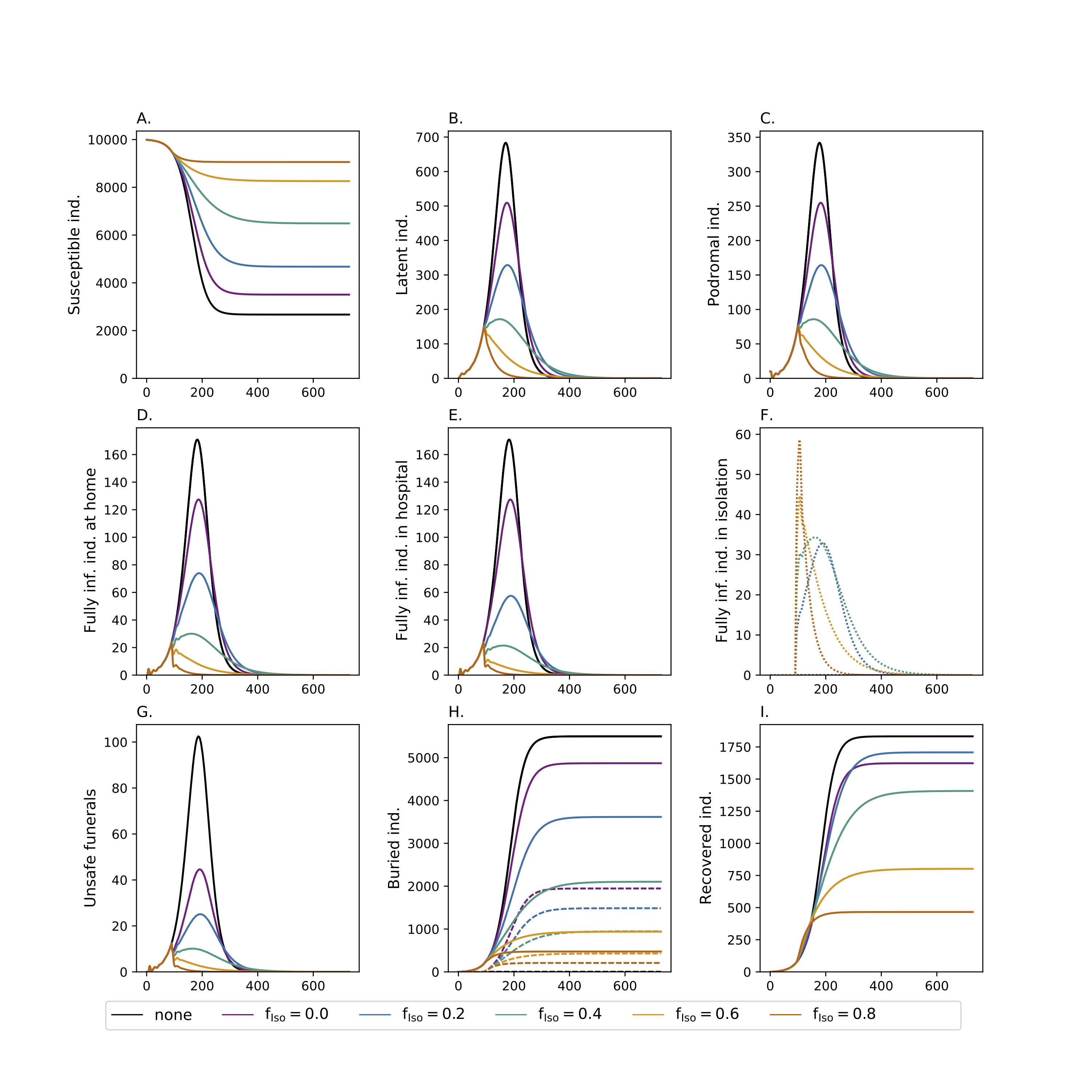

Supplement: S2 Fig — Shown are the same measures as for Fig 3 but under the assumption of severe mortality (see S7 Table). (JPG) [file pone.0276351.s010.jpg]

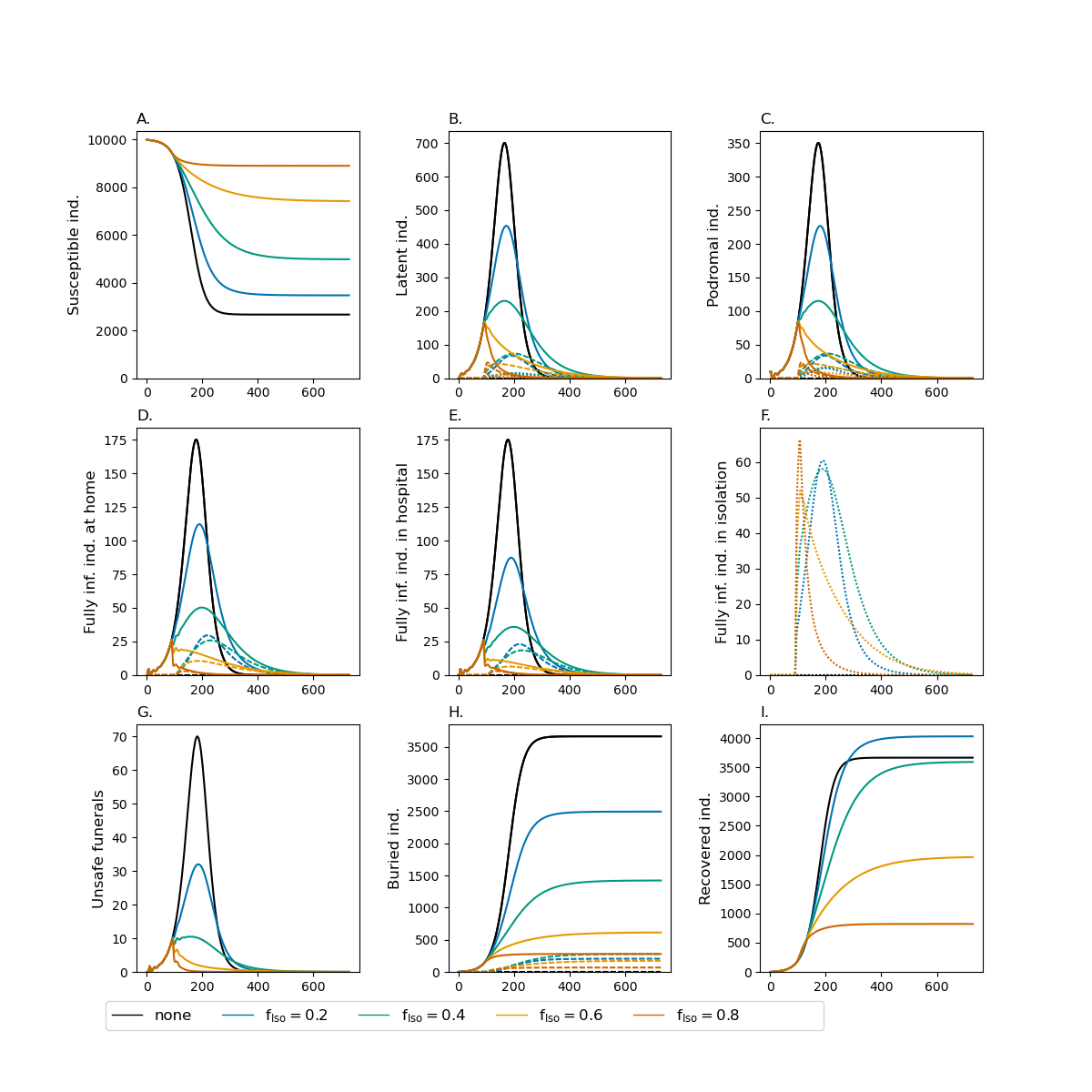

Supplement: S3 Fig — See Fig 2 but combined with additional contact tracing. A fraction fTr = 0.8 of the contacts of infections in isolation are traced back and isolated themselves. In panels (B-F) the dashed lines show the number of infections that will be traced back at some time in the future (not yet isolated) or are currently traced back (and in isolation). The dotted lines show all individuals currently in isolation. In panel (H) the dashed lines show the numbers of safe funerals that were conducted. (PNG) [file pone.0276351.s011.png]

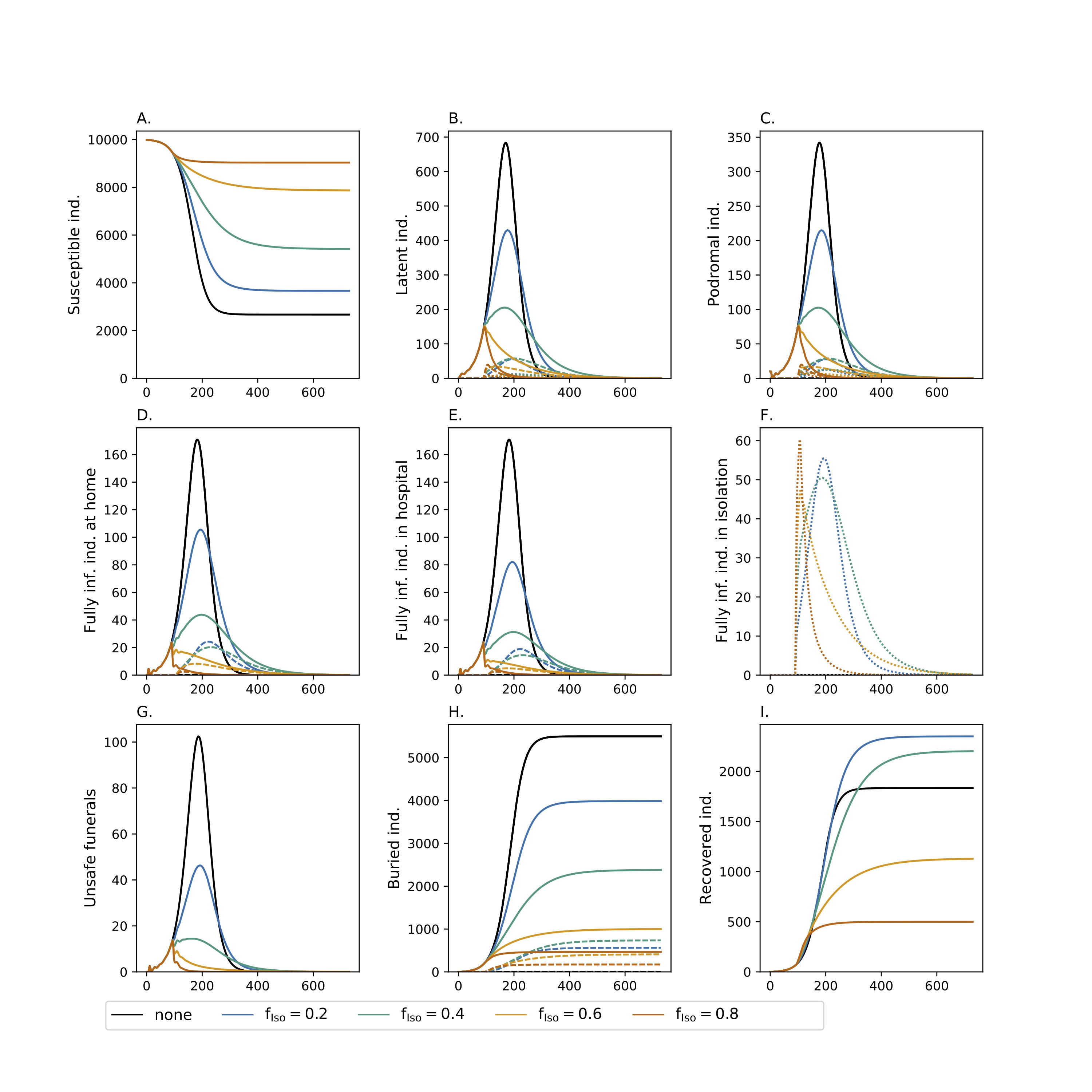

Supplement: S4 Fig — Shown are the same measures as for S3 Fig but under the assumption of severe mortality (see S7 Table). (JPG) [file pone.0276351.s012.jpg]

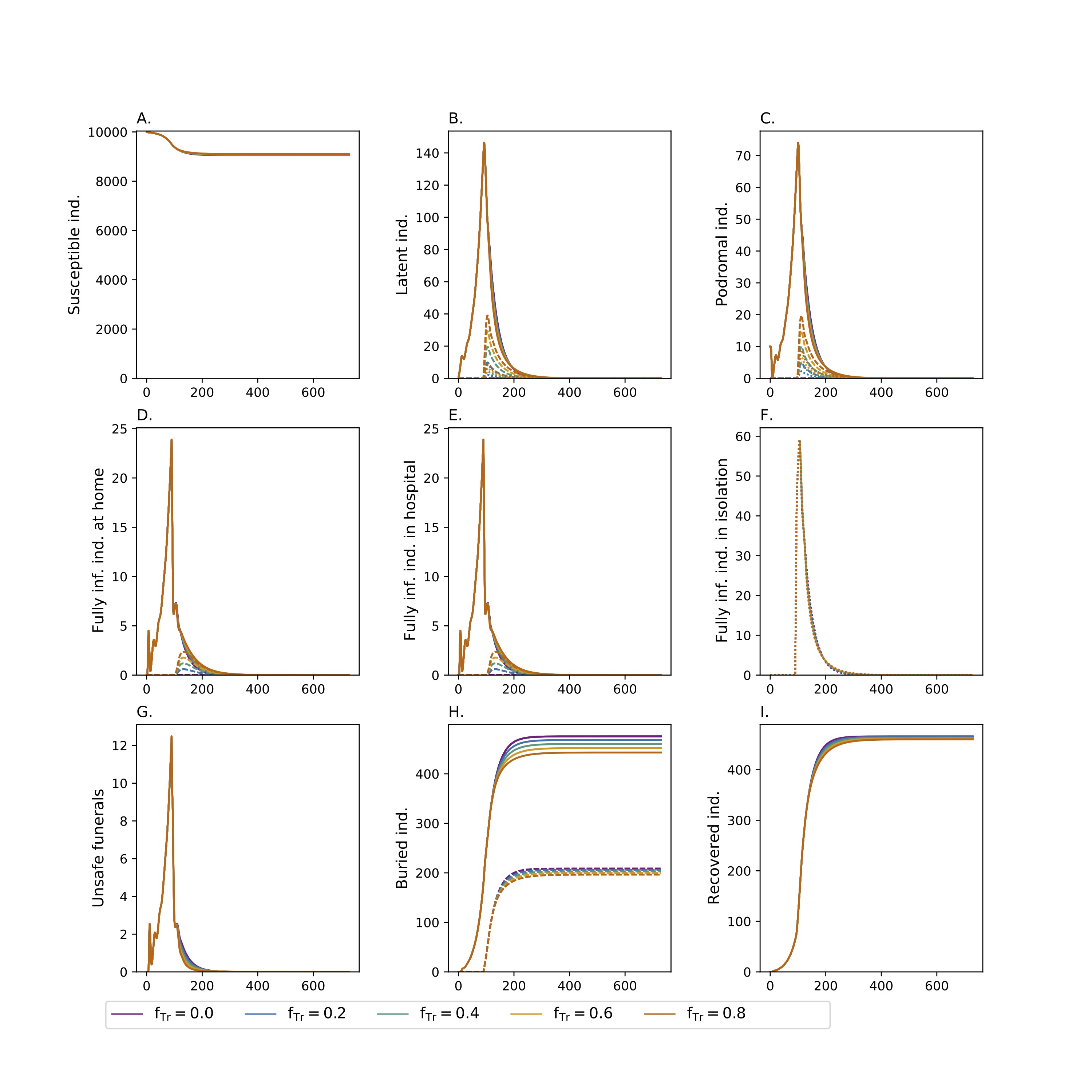

Supplement: S5 Fig — Shown are the same measures as for Fig 4 but under the assumption of severe mortality (see S7 Table). (JPG) [file pone.0276351.s013.jpg]

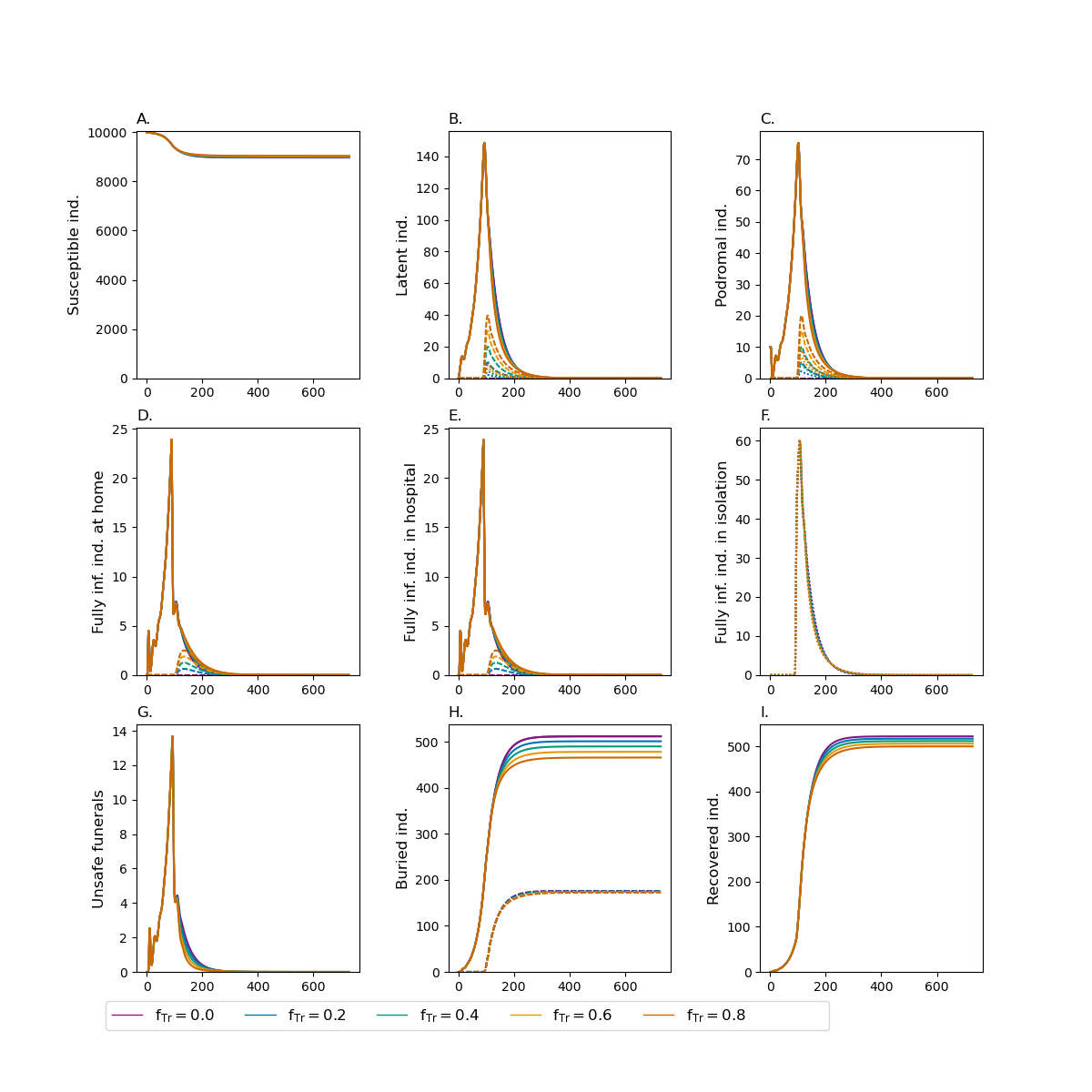

Supplement: S6 Fig — Effect of fraction of infected individuals who will be traced back fTr (colors), when the fraction of infections that are isolated is fIso = 0.8 under the assumption of severe mortality (see S7 Table), but without additional save funeral practices. Line types as in S3 Fig. (PNG) [file pone.0276351.s014.png]

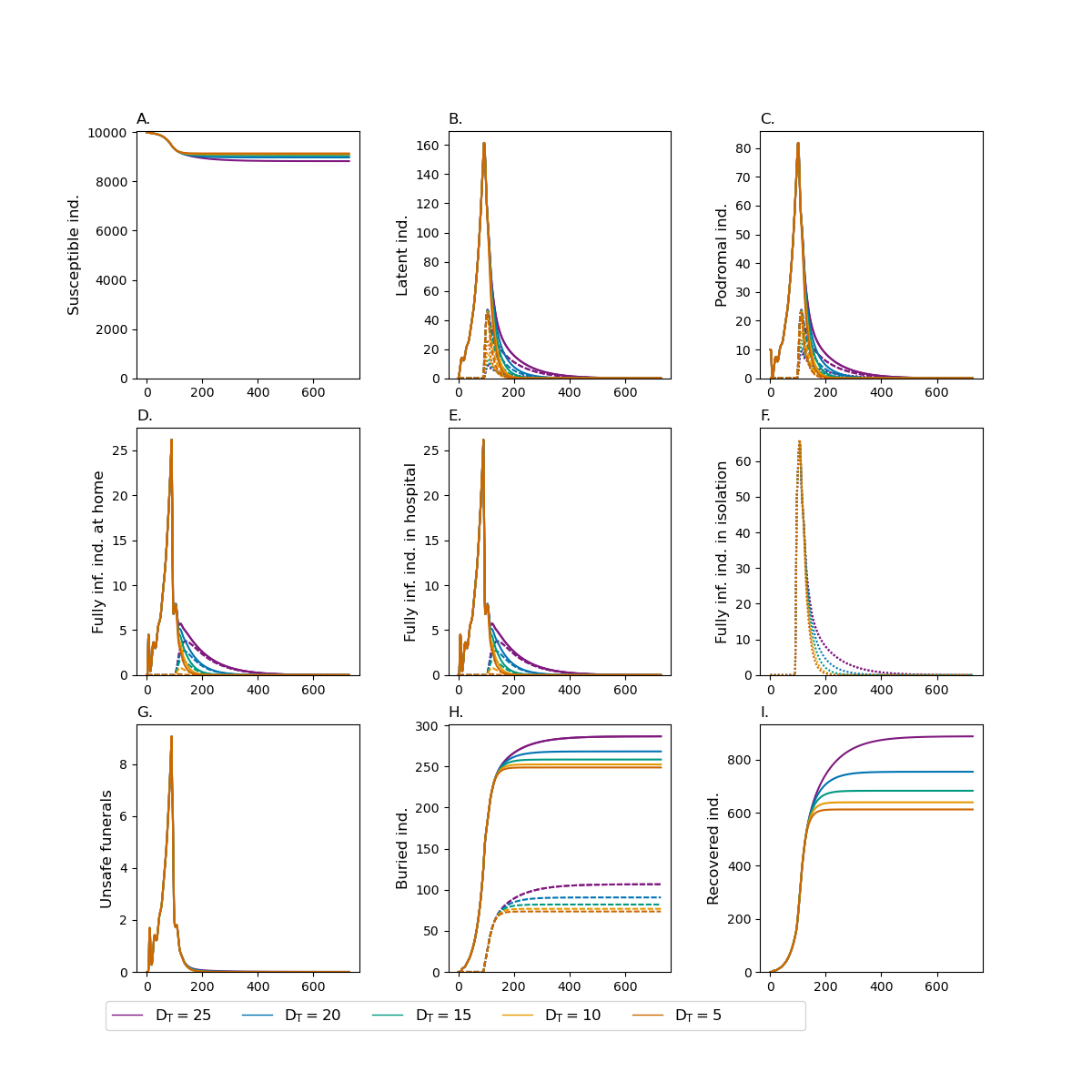

Supplement: S7 Fig — Shown is the effect of the average trace-back time DT (colors), assuming 80% of infections are isolated (fIso = 0.8) and 80% of contacts of isolated persons are traced back (fTr = 0.8). Additional safe funeral practices for lethal cases that occurred outside isolation (dHome = 0.16 and dHosp = 0.8) are assumed. Line types as in S3 Fig. (PNG) [file pone.0276351.s015.png]

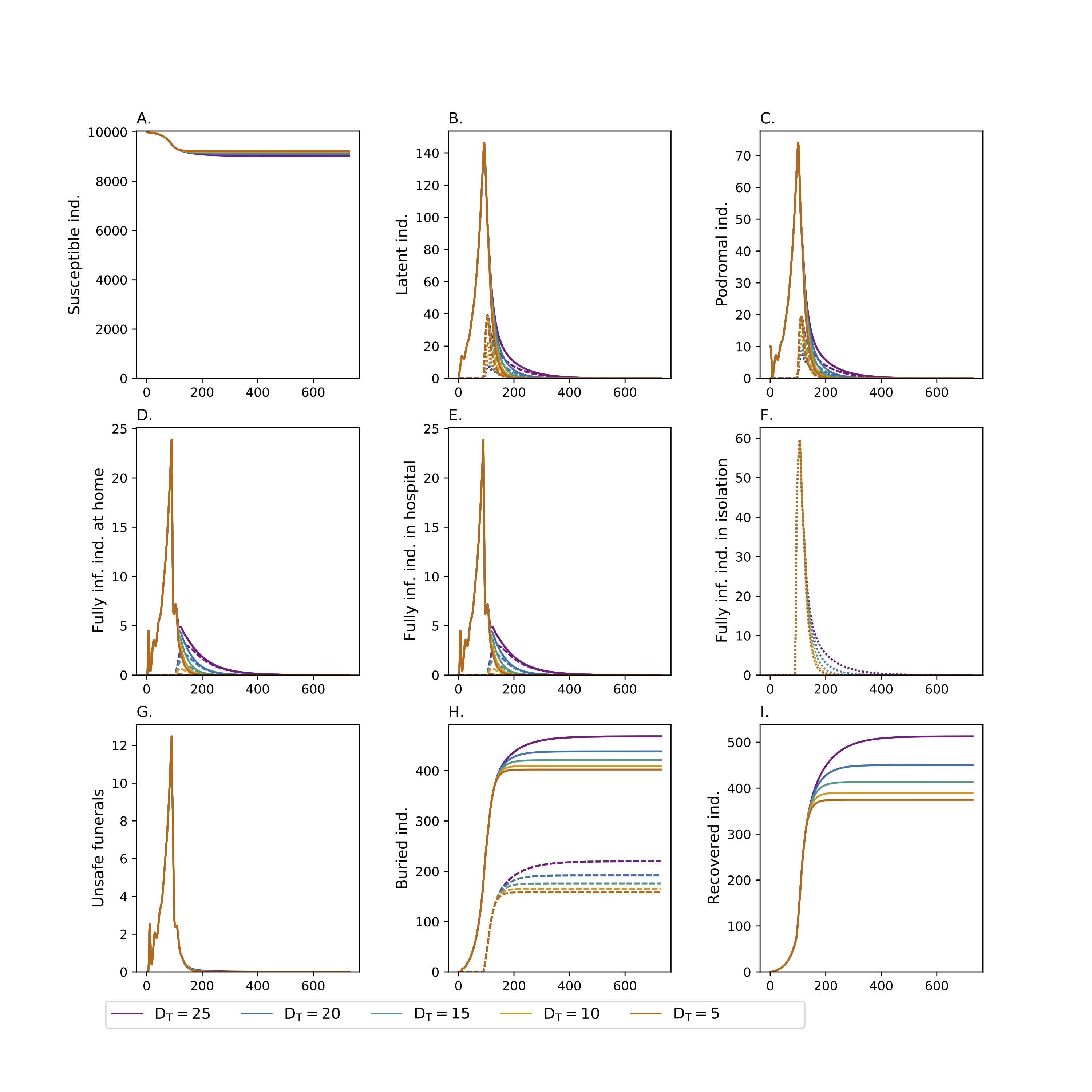

Supplement: S8 Fig — Shown are the same measures as for S7 Fig but under the assumption of severe mortality (see S7 Table). (JPG) [file pone.0276351.s016.jpg]

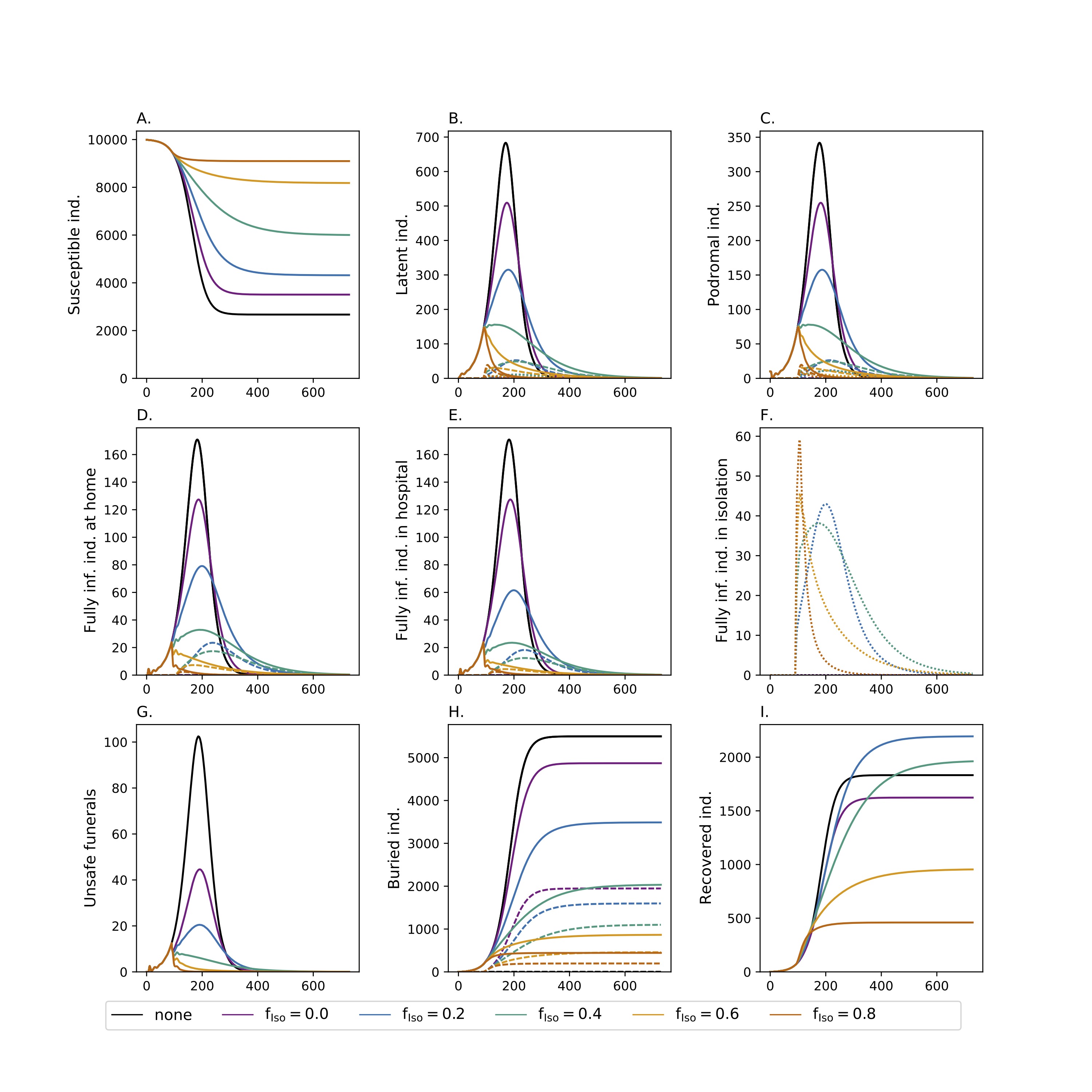

Supplement: S9 Fig — Shown are the same measures as for Fig 5 but under the assumption of severe mortality (see S7 Table). (JPG) [file pone.0276351.s017.jpg]

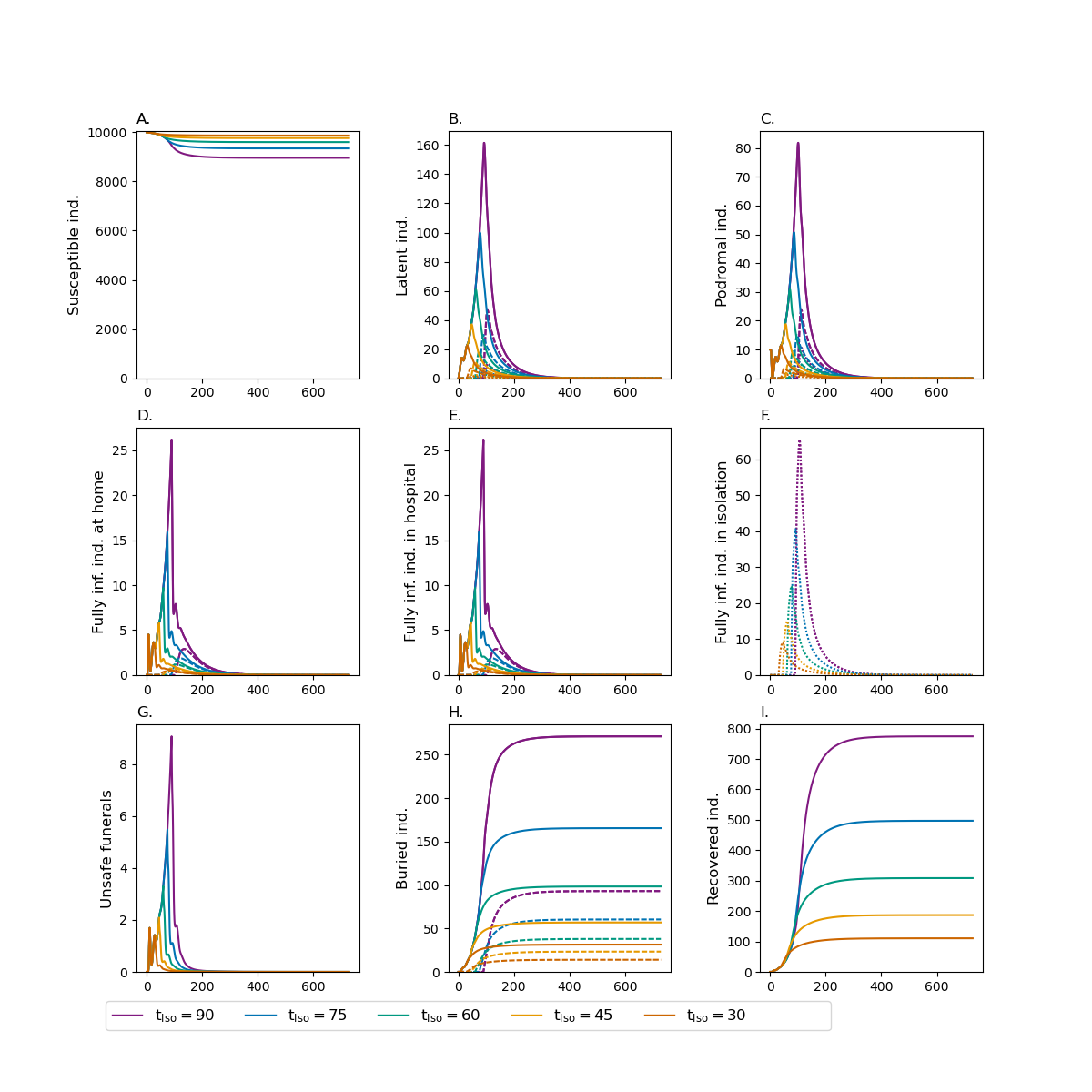

Supplement: S10 Fig — The fraction of infections that are isolated is fIso = 0.8, 80% (fTr = 0.8) of the contacts of isolated patients are subject to back-tracking, and safe funeral practices are conducted outside isolation (dHome = 0.16 and dHosp = 0.8). Line types as in S3 Fig. (PNG) [file pone.0276351.s018.png]

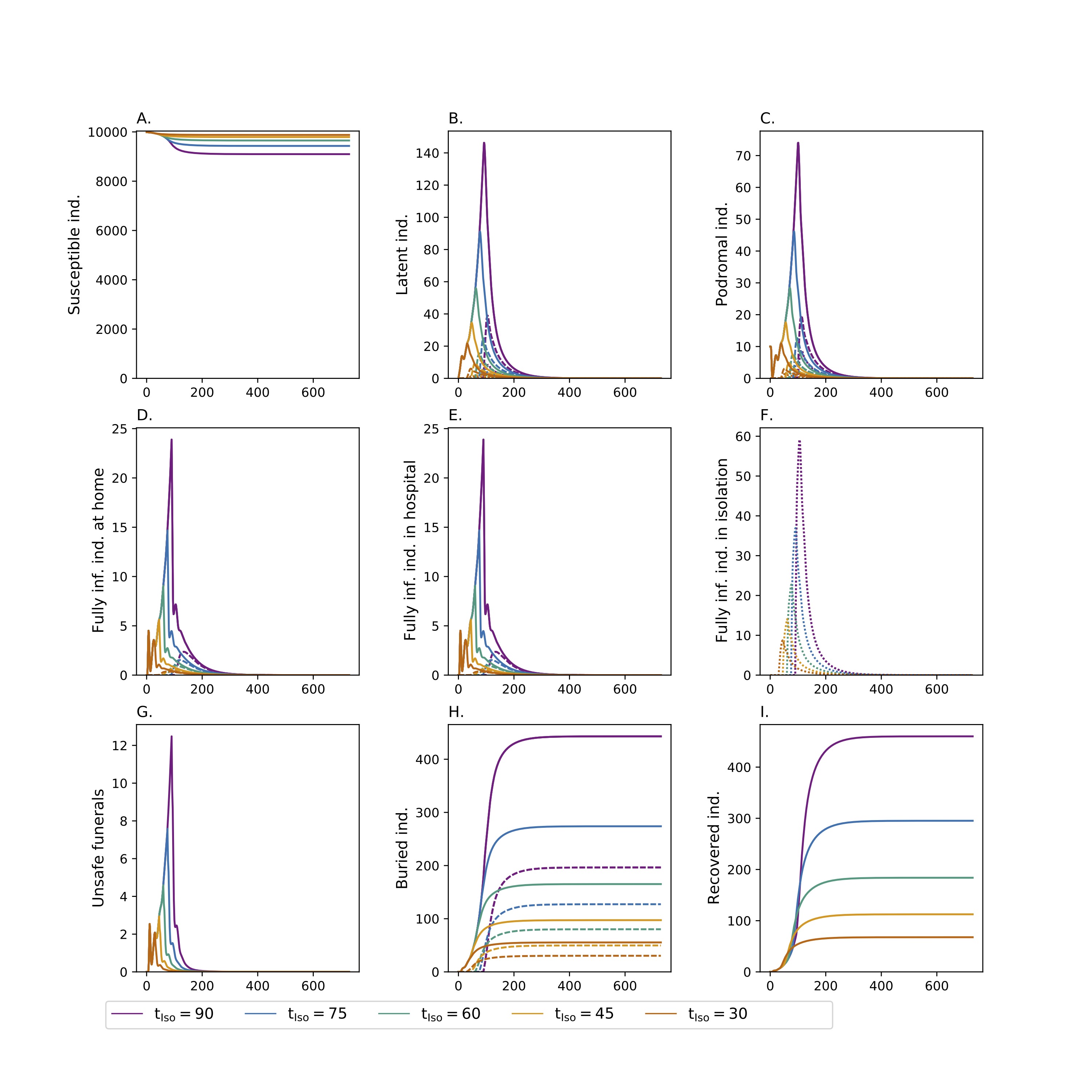

Supplement: S11 Fig — (JPG) [file pone.0276351.s019.jpg]
